# Novel, nanomagnetic and recoverable copper(0)-catalyst in one-pot access to 1,4-disubstituted 1,2,3-triazoles and 5-substituted 1*H*-tetrazoles in water

**DOI:** 10.1098/rsos.241322

**Published:** 2025-02-12

**Authors:** Mohammad Amiri, Nastaran Bagherzadeh, Ali Reza Sardarian

**Affiliations:** ^1^ Chemistry Department, College of Science, Shiraz University, Shiraz, Fars, Iran

**Keywords:** aqueous media, heterogeneous nanocatalyst Cu(0), 1,4-disubstituted-1,2,3-triazoles, 5-substituted 1H-tetrazoles

## Abstract

A novel Fe_3_O_4_@SiO_2_-Pr-2-Py-Cu(0) complex was produced as a heterogeneous magnetic nanocatalyst and characterized by employing several spectroscopic methods such as XRD, FT-IR, BET, VSM, FE-SEM, ICP, TGA, EDX, mapping, and UV-Vis spectroscopy. The catalytic performance of this novel catalyst was examined for efficient access to diverse 1,4-disubstituted-1,2,3-triazoles via eco-friendly one-pot three-component reactions of sodium azide, various terminal alkynes, and benzyl, allyl, ester, or alkyl halide in aqueous media and mild conditions. In addition, catalyst activity to study the generality and scope of the reaction in the library preparation of 5-substituted 1*H*-tetrazoles with a variety of electron-withdrawing and electron-donating groups was used through a one-pot reaction of hydroxylamine hydrochloride, diverse aldehydes and sodium azide in water. Catalyst recyclability results showed that it could operate up to eight consecutive runs without a significant reduction in the reaction efficiency. This green strategy assigns substantial advantages such as efficient and environmentally benign procedures, chemical waste minimization, stability and cost-effectiveness, short reaction time, wide-ranging substrate, an excellent yield of desired products and recoverable and reusable nanomagnetic catalysts.

## 1. Introduction

During the past few decades, vitally important research efforts have focused on improving green and sustainable organic transformation. It is essential to emphasize the heterogenization of super-active catalysts on various inorganic and organic supports, including metal oxide nanoparticles, in order to combine the favoured position of both heterogeneous and homogeneous catalysts [1]. Among various supports and metal oxide nanoparticles, magnetic nanoparticles (MNPs), particularly Fe_3_O_4_ nanoparticles, have shown promise in the field of green nanotechnology due to their high surface area, ease of magnetic removal, simple preparation and functionalization, low toxicity and biocompatibility [2]. However, Fe_3_O_4_ MNPs are prone to aggregation due to anisotropic dipolar attractions. By coating Fe_3_O_4_ MNPs with a biocompatible inorganic protecting layer such as SiO_2_, their stability can be improved. Additionally, the hydroxyl groups of silica on the surface of Fe_3_O_4_@SiO_2_ facilitate bonding with other organic groups, allowing for further chemical modification [3]. Numerous reports have demonstrated the successful application of magnetic core-shell (Fe_3_O_4_@SiO_2_) to immobilize a wide range of organic catalysts, transition metal catalysts and biocatalysts. These catalyst systems have proven to be recyclable, economically viable and eco-friendly for organic transformations [4,5].

Copper-supporting Fe_3_O_4_@SiO_2_ NPs are effective for producing economical and advanced copper nanomaterials with valuable catalytic activity. These nanomaterials enable a wide range of organic reactions, including azide-alkyne cycloaddition, oxidation, reduction, cross-coupling and Ullmann reactions, to be conducted under greener conditions compared with the conventional homogeneous catalysts [6,7]. The discovery of nitrogen-based heterocyclic compounds through green synthesis has garnered significant attention in synthetic and pharmaceutical chemistry. Notably, 1,2,3-triazoles and tetrazoles are well-known structural motifs in this field.

**Figure 1.**
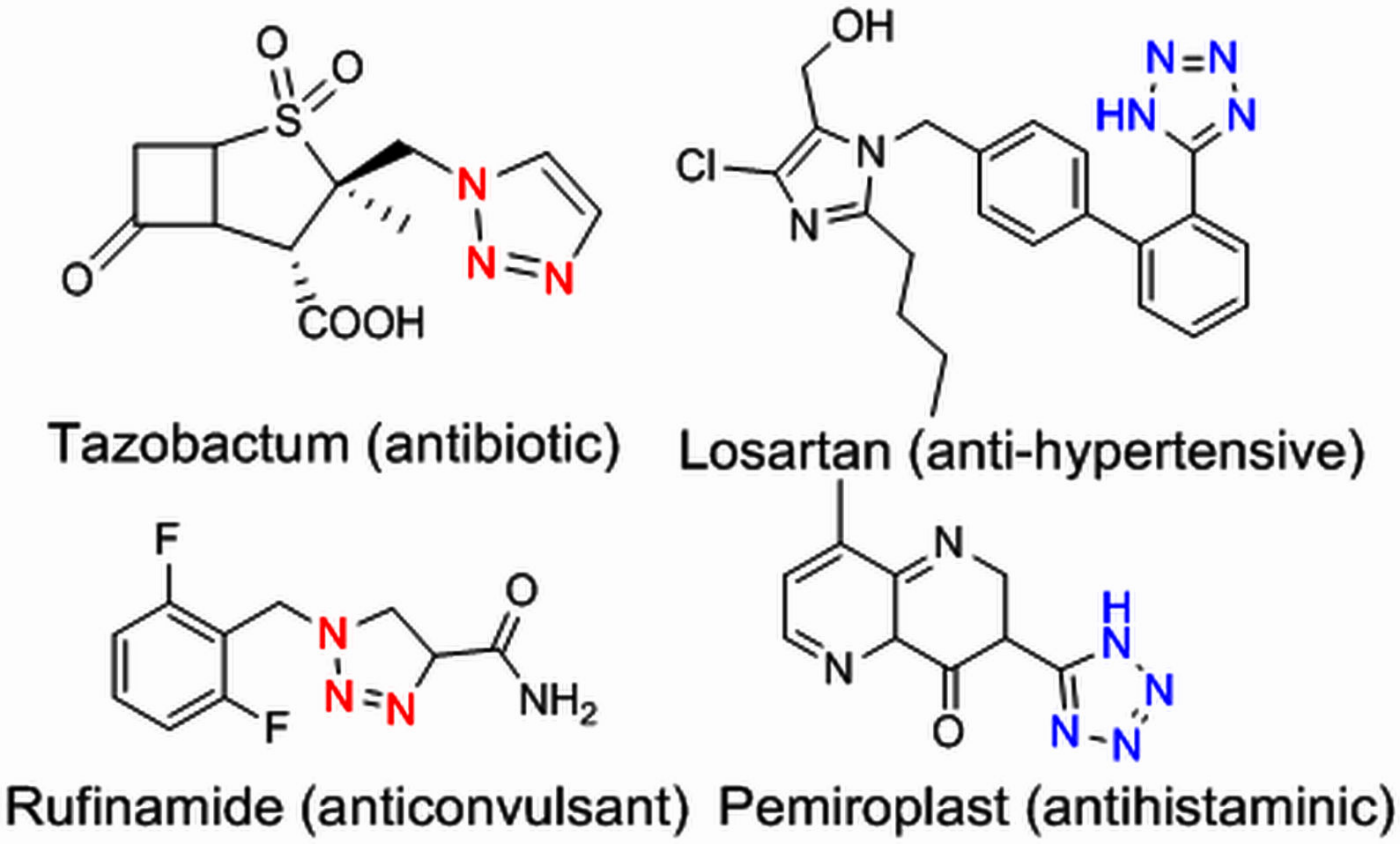
Some active pharmaceutical ingredients comprising a 1,2,3-triazole or tetrazole segment.

Due to their unique structural and chemical properties, 1,2,3-triazole derivatives, which are valuable in medicinal chemistry, exhibit a wide variety of pharmacological and biological activities including anti-influenza, anti-cancer, anti-HIV, anti-epileptic , and cannabinoid receptor antagonist’s activities (figure 1) [8–10]. Additionally, they are applicable in material sciences and industries such as agrochemicals, sensors, dyes, photo stabilizers and corrosion inhibitors [11–15]. Consequently, the formation of 1,4-disubstituted 1,2,3-triazoles utilizing novel, simple, green and efficient methods has drawn the attention of many researchers in synthetic chemistry. The copper-catalysed azide-alkyne cycloaddition (CuAAC) click reaction, which was initially described by Meldal and Sharpless in 2002, is the most renowned method for constructing 1,2,3-triazoles [16–22]. However, due to the challenges associated with working with pre-made organic azides and their explosive properties, *in situ,* the production of organic azides through the reaction of benzyl halide and aryl boronic acid reagents with sodium azide in the presence of a modified catalyst has been reported as an alternative approach to prepare a wide range of these compounds [23–27].

**Scheme 1.**
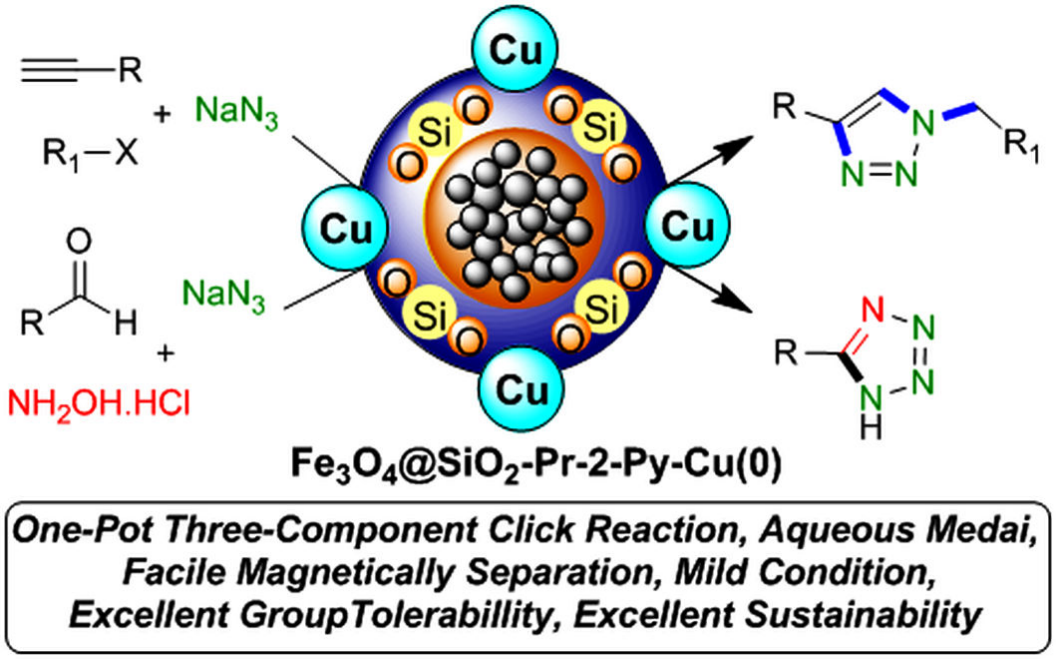
Formation of 1,4-disubstituted-1,2,3-triazoles and 5-substituted 1*H*-tetrazoles by Fe_3_O_4_@SiO_2_-Pr-2-Py-Cu(0) nanocatalyst.

Tetrazole five-membered heterocyclic scaffolds have been widely found in agrochemical products, energetic materials [28], organocatalysis [29,30], coordination and supramolecular chemistry [31] and pharmaceutical drugs as the active pharmaceutical ingredient (API) (such as anti-microbial, anti-cancer, anti-platelets, HIV protease inhibitor and anti-tumour properties) [32,33]. Considerable focus has been dedicated to the effective preparation of tetrazole derivatives in academic and industrial research. Frequently, in the field of medicinal chemistry, the 5-substituted 1*H*-tetrazole motif has been employed as a carboxylic acid isostere [34,35]. Currently, numerous APIs comprising a 1,2,3-triazole or tetrazole segment are available in the pharmaceutical market, as shown in figure 1. According to the reports, there are several prevalent procedures for access to 5-substituted 1*H*-tetrazoles via the reaction of nitriles [36,37], boronic acids [4], and aldehydes [38] with the source of azides such as trimethylsilyl azide (TMSA), tributyltin azide (TBSnA), hydrazoic acid (HN_3_), *p*-toluenesulfonyl azide, benzenesulfonyl azide, diphenyl phosphoryl azide (DPPA), tetrabutylammonium azide (TBAA) and sodium azide (NaN_3_) [39,40]. Among these, the [3+2] cycloaddition of NaN_3_ to nitriles has been efficient and attractive. However, the toxicity and hard biodegradation of most nitriles have encouraged researchers to develop sustainable processes [41,42]. In one of these strategies, aldehydes, which are less toxic, easily available and cheaper, were employed in place of nitriles [43], but using high-boiling-point organic solvents, harsh reaction conditions, expensive metal salts and homogeneous catalysts in the reaction process confines their utilization in biochemical research. As a result, developing procedures compatible with aqueous and environmentally benign conditions in the presence of heterogeneous green nanocatalysts remains a challenge. Therefore, in this context, we design a strategy to access a variety of 1,4-disubstituted 1,2,3-triazoles and 5-substituted *1H*-tetrazoles in water under mild reaction conditions by synthesizing a novel Cu(0) complex-based superparamagnetic heterogeneous nanocatalyst, Fe_3_O_4_@SiO_2_-Pr2-Py-Cu(0) using pyridine-2-carbaldehyde as an organic ligand (scheme 1).

## 2. Material and methods

### 2.1. Chemical and instrumentation

In this project, all starting materials and reagents were obtained from reputable suppliers such as Across Chemicals, Merck and Sigma-Aldrich ensuring high purity. Thin layer chromatography (TLC) on silica sheets 60 F254 was utilized to determine the reaction progress and assess the purity of the substances. The Shimadzu FT-IR 8300 model spectrometer was used to obtain FT-IR measurements using KBr pellets in the range of 400 to 4000 cm^−1^. The NMR spectra were acquired using Bruker Avance DPX400 spectrometers at 400 MHz for ^1^H NMR and 101 MHz for ^13^C NMR in CDCl_3_ and DMSO-*d_6_*. Fe_3_O_4_@SiO_2_-Pr-2-Py-Cu(0) nanoparticles were studied using X-ray diffraction (XRD) on a Bruker AXS D8‐Advance X‐ray diffractometer with CuKα radiation (λ = 1.5418 Å). In order to observe the morphology of the nanocatalyst, field emission scanning electron microscopy (FE‐SEM) on the MIRA3 instrument was utilized to see the spherical shape and size of the catalyst. A VASCO nanoparticle size analyser was used to measure dynamic light scattering (DLS). Transmission electron microscopy (TEM) was performed using a Zeiss electron microscope with an acceleration voltage of 100 kV. FE-SEM was characterized using the MAIA3 model. A UV-Vis spectrophotometer (PerkinElmer, Lambda 25) was utilized to record UV-Vis absorption spectra. A vibrating sample magnetometer (VSM) (instrument model BHV-55) was used for characterization. The BET surface areas were obtained using the Autosorb-iQ-MP, an automatic analyser manufactured by Quantachrome in the USA; this was achieved by determining nitrogen desorption–adsorption isotherms at a temperature of liquid N_2_. The nanocatalyst elements were determined using energy-dispersive X-ray (EDX) spectroscopy in conjunction with a TESCAN-Vega 3 model scanning electron microscope (SEM). The copper loading on the supporting materials was detected using an ICP analyser, specifically the Varian Vista-pro model. A PerkinElmer device was applied to determine thermogravimetric analysis (TGA) data. Melting points were measured using a micro melting point apparatus called Electrothermal, BUCHI 510. A Thermo Finnigan Flash EA-1112 CHNS rapid elemental analyser was applied to carry out elemental analyses (C, H, N). Eventually, the comparison of the ^1^H NMR and ^13^C NMR spectra and the melting points of the products was conducted with literature values.

### 2.2. General procedure

#### 2.2.1. (1-(Pyridine-2-yl)-*N*-(3-(trimethoxysilyl)propyl) methanimine) (L1) preparation

**L1** was constructed according to the method described earlier [44].

#### 2.2.2. Fe_3_O_4_ and Fe_3_O_4_@SiO_2_ NP syntheses

These NPs were synthesized according to the previous procedure developed by our group [4].

#### 2.2.3. 1-(Pyridine-2-yl)-*N*-(3-(trimethoxysilyl)propyl)methanimine-magnetic-silica) (L2) preparation

The synthesized Fe_3_O_4_@SiO_2_ (1 g) was added to 20 ml toluene and dispersed for 15 min. After adding 5 mmol (1.34 g) of the ligand L1, the mixture was refluxed for 24 h under a N_2_ atmosphere. Afterwards, it was permitted to reach the ambient temperature. Then the nanoparticles collected by an external magnet were washed twice with 10 ml of EtOH and dried at 80°C [45].

#### 2.2.4. *N*-(Pyridine-2-ylmethyl)propylamino magnetic-silica (L3) preparation

To a 25 ml round-bottom flask, 15 ml absolute ethanol was added to 1 g of ligand L2. Then, the flask was transferred to an ice bath to cool the reaction mixture to 0°C. After some time, 5.02 mmol (0.190 g) of NaBH_4_ was slowly added to the mixture while stirring, and let to reach the ambient temperature for 5 min. The resulting mixture was stirred at ambient temperature for 24 h. Afterwards, the reduced nanocatalyst is collected using an external magnet, and the final mixture is washed with water and ethanol to obtain the product in a 92% yield [45].

#### 2.2.5. Fe_3_O_4_@SiO_2_-Pr-2-Py-Cu complex (L3–Cu) preparation

To a dispersed solution of 0.5 g ligand L3 in 10 ml absolute ethanol was added 3.76 mmol (0.75 g) of Cu(OAc)_2_.H_2_O. Then, the mixture was stirred under reflux for 6 h. The L3–Cu complex was then completely separated from the reaction using an external magnet bar. To complete the process, the collected precipitate was washed with ethanol and distilled water and dried for 5 h at 60°C, resulting in a 95% yield of L3–Cu.

#### 2.2.6. General production of propargyl-phenol ethers

Phenol (1 mmol, 94 mg) and K_2_CO_3_ (2 mmol, 198 mg) were added to 5 ml of acetone. After stirring the mixture at room temperature for 15 min, propargyl bromide (1.5 mmol, 1.77 mg) was added dropwise and the mixture was heated under reflux for 3 h. The mixture was then quenched with 10 ml of water and evaporated under reduced pressure. The residue aqueous layer was extracted twice with 10 ml of ethyl acetate. The combined organic phase was dried over anhydrous Na_2_SO_4_. It was then evaporated in a vacuum, and the residue was moved to the next stage without purification [46].

#### 2.2.7. General production of 1,4-disubstituted-1,2,3-triazoles

To synthesize title 1,2,3-triazoles, Fe_3_O_4_@SiO_2_-Pr-2-Py-Cu(0) (0.25 mol% Cu, 10 mg) is poured into the blend of NaN_3_ (1.5 mmol), terminal alkyne (1.2 mmol) and alkyl/aryl halide (1 mmol) in 4 ml H_2_O stirring. The blend is heated at 60°C for the appropriate session, which is determined by TLC checking. After the completion of the reaction, the catalyst is effectively separated by applying an external magnet and the residue is extracted threefold with the mixture of ethyl acetate and water (1:1). The dried organic phase with anhydrous Na_2_SO_4_ is concentrated under vacuum. The isolated impure product is refined by performing silica gel column chromatography with a mixture of *n*-hexane and ethyl acetate in a ratio of 5:2 or by washing twice with cold diethyl ether.

#### 2.2.8. General production of 5-substituted 1*H*-tetrazoles

Fe_3_O_4_@SiO_2_-Pr-2-Py-Cu(0) (0.4 mol% Cu, 16 mg) is added as a catalyst to the blend of H_2_O (5 ml), NaN_3_ (1.5 mmol, 98 mg), aldehyde (1.0 mmol) and hydroxylamine HCl (1.5 mmol, 104 mg). The mixture is then stirred under reflux conditions for the proper time. Once the reaction is complete (monitored by TLC), the mixture is cooled to ambient temperature. The Cu(0)-nanocatalyst is collected using an intense external magnetic field, and the remaining mixture is treated with 5 M HCl (10 ml) while being vigorously stirred for 5 min. The aqueous solution is then extracted with ethyl acetate (2 × 10). The extracted organic phase is dried using anhydrous Na_2_SO_4_ and concentrated under a vacuum to provide the crude product, which is refined through crystallization with diethyl ether.

## 3. Results and discussion

**Scheme 2.**
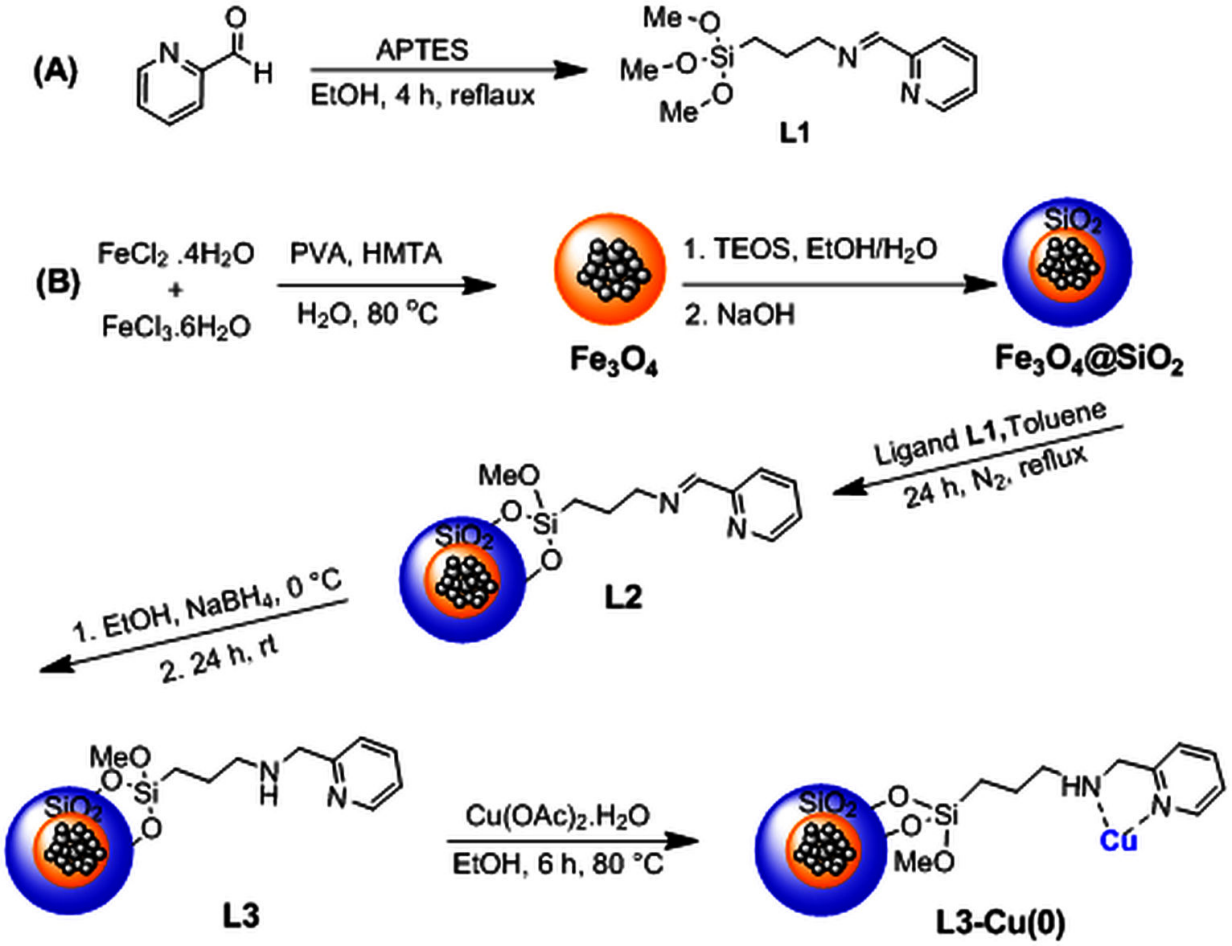
Production approach of L3-Cu nanocatalyst.

Synthetic steps of this superparamagnetic nanocatalyst bearing Cu(0) particles (Fe_3_O_4_@SiO_2_-Pr-2-Py-Cu(0)) are summarized in scheme 2.

**Figure 2.**
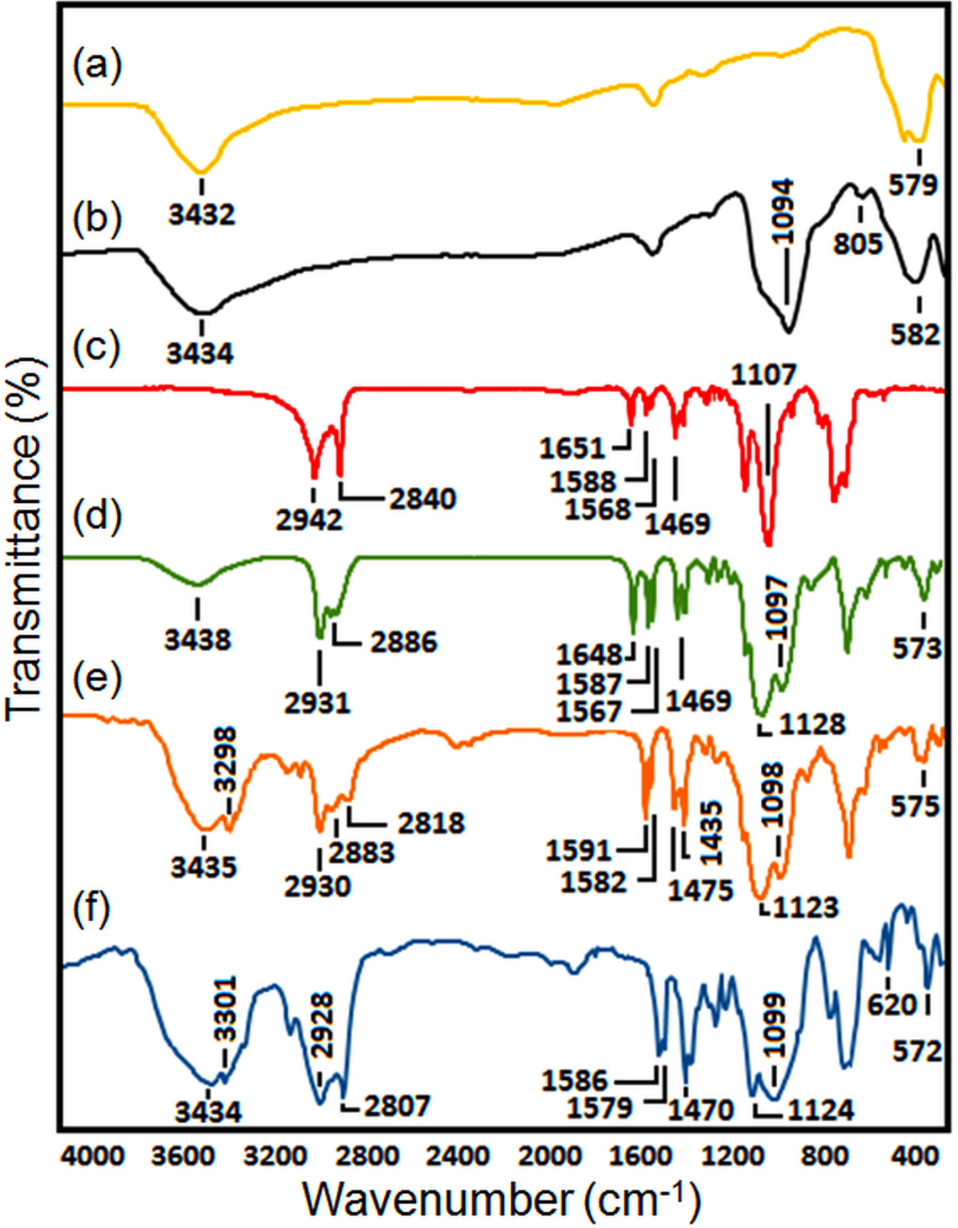
FT-IR spectra: (*a*) Fe_3_O_4_, (*b*) Fe_3_O_4_@SiO_2_, (*c*) Schiff-base, (*d*) Fe_3_O_4_@SiO_2_-Schiff-base, (*e*) Fe_3_O_4_@SiO_2_-Pr−2-Py, and (*f*) Fe_3_O_4_@SiO_2_-Pr−2-Py-Cu(0).

To investigate the different functional groups in the synthesized magnetic nanocatalyst, we conducted FT-IR spectroscopic analysis of the catalyst and its precursors. In figure 2*a*, the specific band at 579 cm^−1^ and 3432 cm^−1^ indicates the Fe–O and O–H stretching vibrations in the tetrahedral site of Fe_3_O_4_. In figure 2*b*, it can be observed that the absorption peaks at 802 cm^−1^ and 1087 cm^−1^ are indicative of the symmetric and asymmetric stretching vibrations of the Si–O–Si bond. This observation justified the effective coating of silica on the outer surface of Fe_3_O_4_ NPs [4]. The successful synthesis of ligand L1 is verified with the broadband at around 1107 cm^−1^, which is related to Si–O of methoxy silane groups, and the specific band at 1651 cm^−1^ due to the absorption of Schiff base (C=N) (figure 2*c*). These results are attributed to the reaction between the carbonyl group of 2-pyridinecarboxaldehyde and the amine group of 3-aminopropyltrimethoxysilane. Furthermore, the absorption peaks observed at approximately 1588 cm^−1^ and 1469 cm^−1^ correspond to the C=N and C=C bonds, respectively, present in the pyridine rings. Additionally, several bands within the range of 2840−2942 cm^−1^ are associated with the stretching of the C–H bonds in the methylene groups of propyl. Moving to figure 2*d*, which shows the FT-IR spectrum of the Fe_3_O_4_@SiO_2_-Schiff base, we observe peaks from both ligand L1 and Fe_3_O_4_@SiO_2_, confirming the successful anchoring of the ligand onto the magnetic core-shell surface. Disappearance of the peak at 1648 cm^−1^ (C=N) and appearance of the NH stretching peak at 3298 cm^−1^ in the FT-IR spectrum of Fe_3_O_4_@SiO_2_-Pr-2-Py (figure 2*e*) reveal the successful reduction of Fe_3_O_4_@SiO_2_-Schiff-base. Finally, in figure 2*f*, the formation of the complex with copper acetate is evidenced by the emergence of a new peak at 620 cm^−1^, which is assigned to Cu–N [47]. Additionally, the shifting of the peaks associated with C=N pyridine rings and N–H, from 1591 cm^−1^ and 3298 cm^−1^ to 1586 cm^−1^ and 3301 cm^−1^, respectively, provides further evidence of the complex formation.

**Figure 3.**
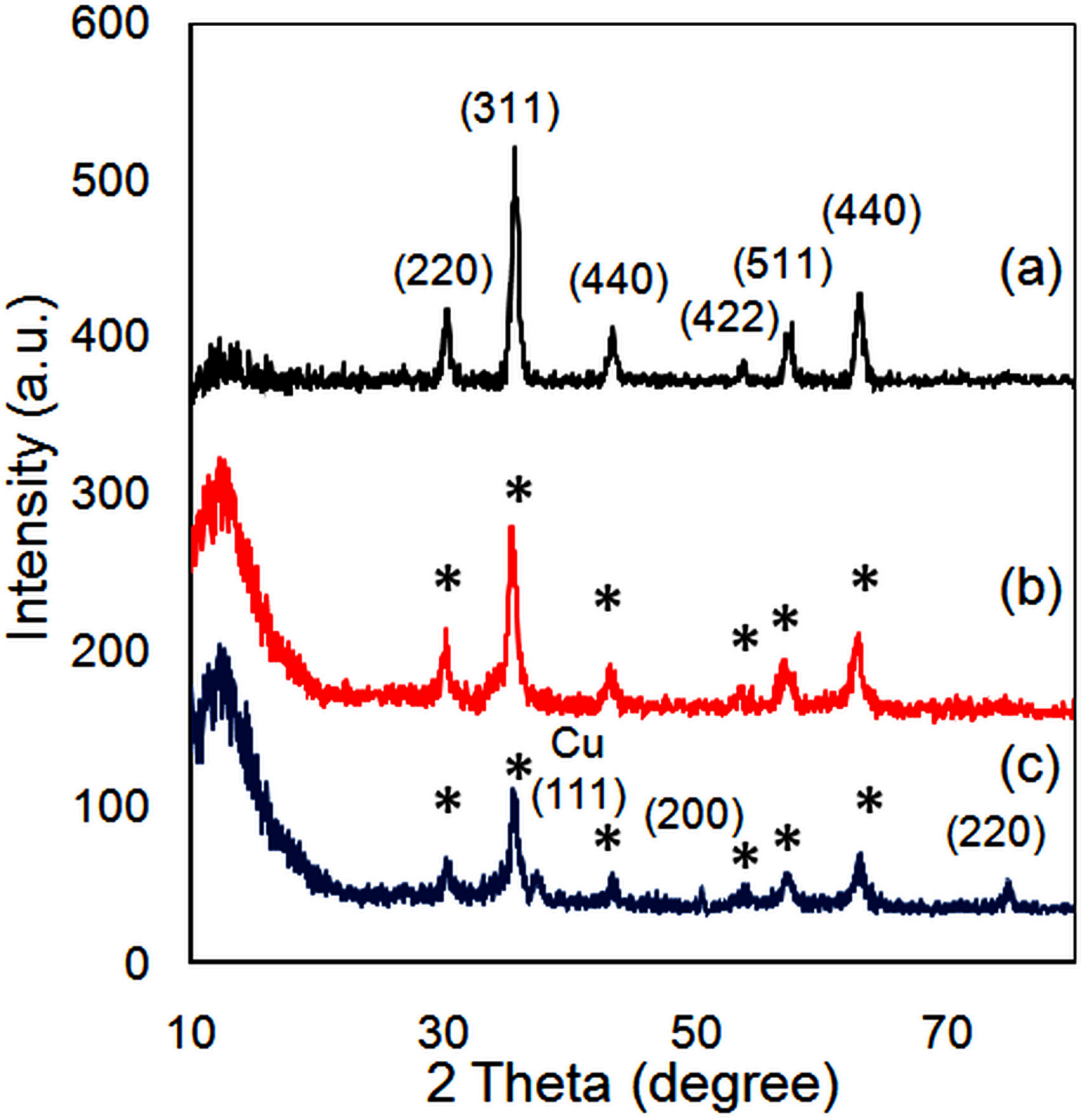
XRD diagrams of (*a*) Fe_3_O_4_, (*b*) Fe_3_O_4_@SiO_2_ , and (*c*) Fe_3_O_4_@SiO_2_-Pr−2-Py-Cu(0).

The XRD patterns of Fe_3_O_4_, Fe_3_O_4_@SiO_2_ , and Fe_3_O_4_@SiO_2_-Pr-2-Py-Cu(0) also demonstrate the successful synthesis of the catalyst (figure 3). Figure 3*a* shows the XRD pattern with diffraction peaks at approximately 2Ө = 30.1, 35.5, 43.1, 53.4, 57.0 and 62.6°, which correspond to the Miller index (220), (311), (400), (422), (511), and (440), respectively. These peaks can be attributed to the standard XRD data of Fe_3_O_4_ NPs with a cubic structure (reference JCPDS card no. 19-629). The reflection intensity of the magnetite phase decreased significantly after it was coated with TEOS. The presence of the amorphous silica phase surrounding the magnetic core was confirmed by the broad peaks observed in figure 3*b*. The XRD pattern of Fe_3_O_4_@SiO_2_-Pr-2-Py-Cu(0) demonstrated three index peaks for copper for 2θ at 42.3, 50.9 and 75.6^o^ for the respectively marked indices of (111), (200) and (220). These characteristic peaks confirm the formation of a face-centred cubic (FCC) copper phase without significant oxides or other impurity phases (figure 3*c*) [48]. The XRD patterns of Fe_3_O_4_@SiO_2_ and Fe_3_O_4_@SiO_2_-Pr-2-Py-Cu(0) NPs exhibited the same diffraction peaks as bare Fe_3_O_4_ nanoparticles, indicating that the surface modification of the Fe_3_O_4_ nanoparticles did not cause any phase change. The average size of the crystallites was determined using Scherrer’s equation, which takes into account a constant value (*K* = 0.94), the full-width at half-maximum (FWHM) in radians (β), the average diameter in Angstroms (D), the wavelength of the X-rays emitted by the anode (1.54 Å for Cu) and the Bragg diffraction angle (Ө). The calculated sizes of the magnetic nanoparticles using the Scherrer equation were 18.6 nm for Fe_3_O_4_, 24.5 nm for Fe_3_O_4_@SiO_2_ and 31.1 nm for Fe_3_O_4_@SiO_2_-Pr-2-Py-Cu(0).

The BET surface area of Fe_3_O_4_@SiO_2_-Pr-2-Py-Cu(0) was determined by analysing N_2_ adsorption isotherms at 77 K. The results revealed an average pore diameter of approximately 29.47 nm. The total pore volume obtained was 0.13 cm^3^ g^−1^, while the specific BET surface area was measured to be 17.59 m^2^ g^−1^.

**Figure 4.**
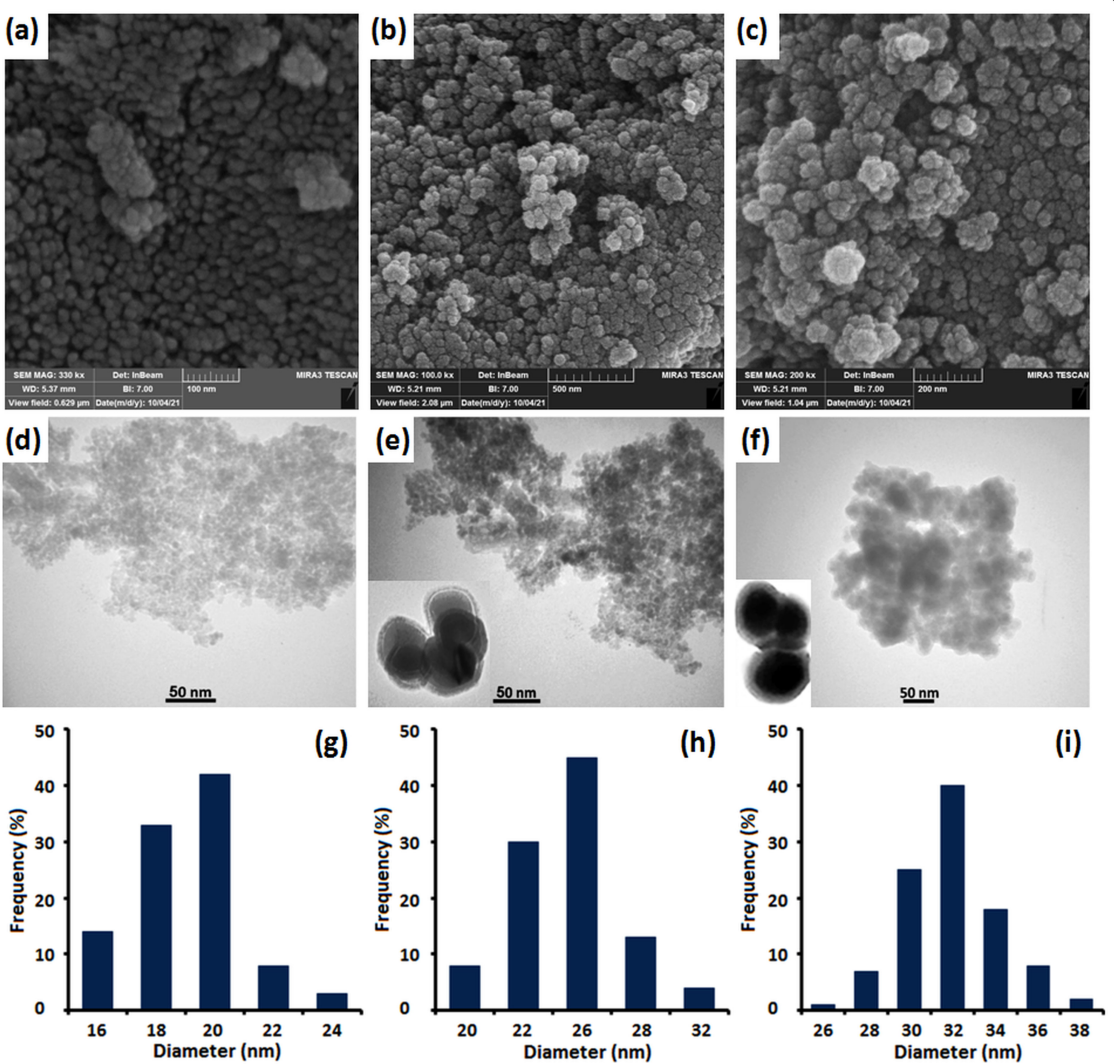
FE-SEM pictures of (*a*) Fe_3_O_4_, (*b*) Fe_3_O_4_@SiO_2_ and (*c*) Fe_3_O_4_@SiO_2_-Pr-2-Py-Cu(0); TEM pictures of (*d*) Fe_3_O_4_, (*e*) Fe_3_O_4_@SiO_2_ and (*f*) Fe_3_O_4_@SiO_2_-Pr-2-Py-Cu(0) and the size distributions of (*g*) Fe_3_O_4_ (*h*) Fe_3_O_4_@SiO_2_ and (*i*) Fe_3_O_4_@SiO_2_-Pr-2-Py-Cu(0).

To investigate the morphology, size and surface structure of the magnetic nanocatalyst, we employed FE-SEM and TEM analysis in a stepwise manner. The obtained Fe_3_O_4_ nanoparticles were spherical, with an average diameter of around 20 nm (figures 4*a*, and *d*). Figure 4*e* revealed the core-shell structure of Fe_3_O_4_@SiO_2_, with a silica shell thickness of around 3.5 nm. Furthermore, figure 4*f* depicts the structure of Fe_3_O_4_@SiO_2_-Pr-2-Py-Cu(0) after ligand coating and complex formation, with a diameter of approximately 32 nm. The FE-SEM images in figures 4*a*–*c* demonstrate that the spherical structure and morphology of the magnetic nanoparticles remained unchanged during the catalyst preparation process. DLS analysis, presented in figures 4*g*–*i*, yielded a distribution histogram of the MNPs, revealing that the average dimensions of the Fe_3_O_4_, core-shell Fe_3_O_4_@SiO_2_ and Fe_3_O_4_@SiO_2_-Pr-2-Py-Cu(0) nanocatalyst were approximately 20 nm, 26 nm and 32 nm, respectively.

**Figure 5.**
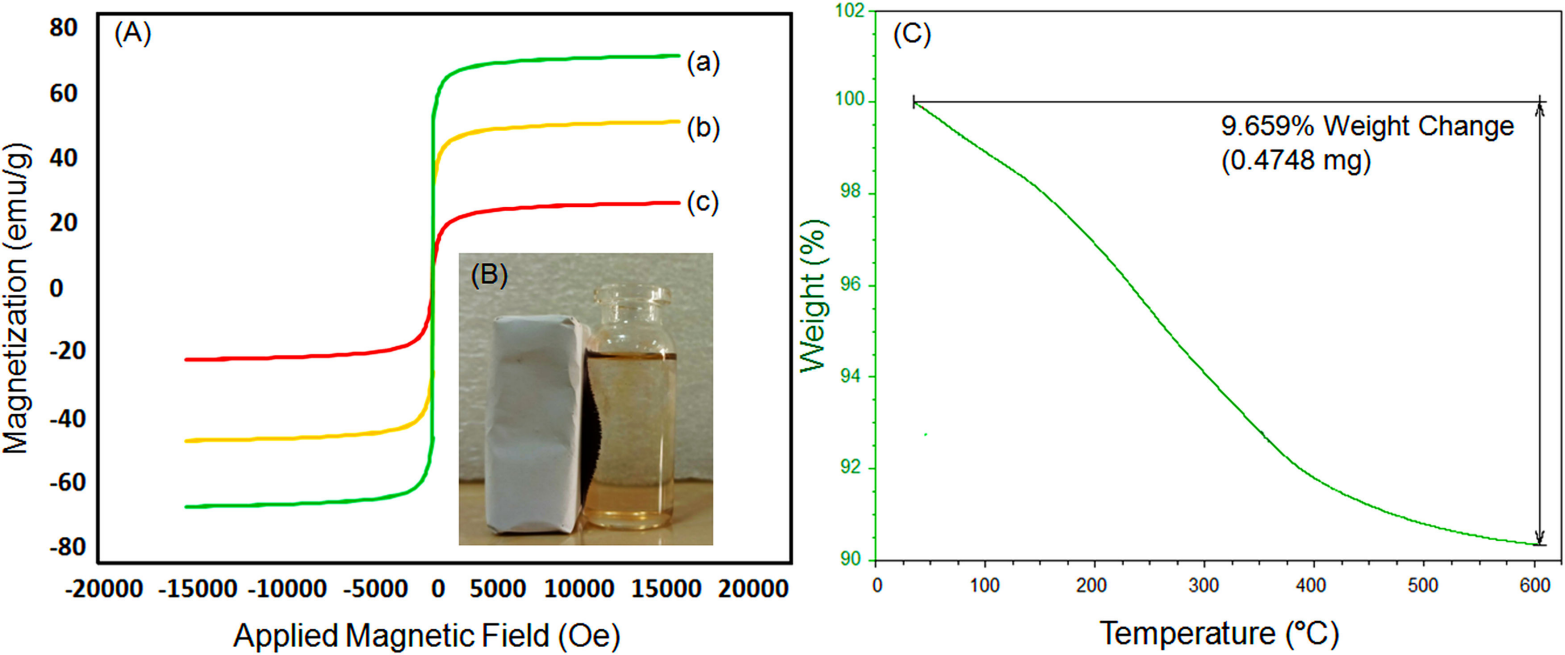
(A) Magnetic hysteresis curves of (*a*) Fe_3_O_4_, (*b*) Fe_3_O_4_@SiO_2_ , and (*c*) Fe_3_O_4_@SiO_2_-Pr-2-Py-Cu(0) NPs; (B) catalyst’s capacity for efficient recovery using an external magnet after the reaction completion; (C) TGA diagram of Fe_3_O_4_@SiO_2_-Pr-2-Py-Cu(0) NPs.

VSM analysis was examined as one of the most important analyses for investigating the magnetic properties of magnetic nanocatalysts, which led to two important results. First, the saturation magnetic values were 66.27, 47.15 and 24.15 emu g^−1^ for the MNPs Fe_3_O_4_, Fe_3_O_4_@SiO_2_ and Fe_3_O_4_@SiO_2_-Pr-2-Py-Cu(0), respectively (figure 5*a*). This decrease in saturation magnetization value in the final catalyst is due to the functionalization of the surface Fe_3_O_4_ MNPs via an organic non-magnetic layer. But despite this reduction, the final catalyst is still well separated from the reaction mixture by a supermagnet (figure 5*b*). Second, the results show that after removing the magnetic field, the catalyst loses its magnetic property, which is evidence that the catalyst is paramagnetic.

One of the salient features of heterogeneous catalysts is their high thermal stability. To investigate the thermal stability of the synthesized Cu(0)-nanocatalyst, TGA analysis was conducted over a temperature range of 25–600°C. As shown in figure 5*c*, the thermogram shows a mass loss in two stages. The initial weight loss in the temperature range of 25 to 140°C is usually related to removing physically adsorbed water and solvent molecules. The second weight loss occurs within the temperature range of 140 to 600°C, which is allocated to the decomposition of the organic layer supported on the catalyst’s surface.

**Figure 6.**
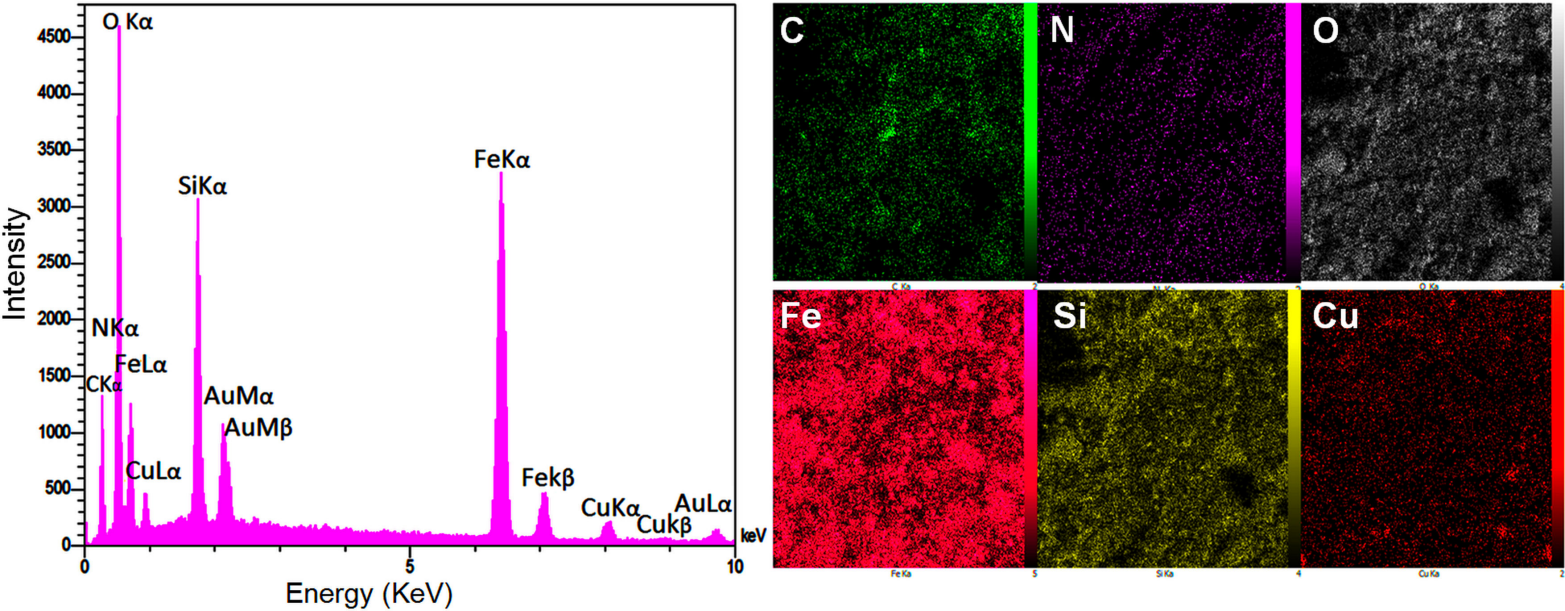
EDX spectra of Fe_3_O_4_@SiO_2_-Pr-2-Py-Cu(0) , and elemental mapping of Fe_3_O_4_@SiO_2_-Pr-2-Py-Cu(0).

EDX and elemental mapping image analysis were carried out to determine the existence of elements in the Fe_3_O_4_@SiO_2_-Pr-2-Py-Cu(0) catalyst (figure 6). The presence of iron (Fe), oxygen (O) and silicon (Si) in the EDX spectrum discloses the presence of the core shell as a supermagnetic substrate. Besides, the three elements nitrogen (N), carbon (C) and copper (Cu) confirm the successful functionalization, ligand attachment and metal complex formation on the surface of the silica-coated magnetite nanoparticle, thus verifying the structure of the magnetic nanocatalyst.

**Figure 7.**
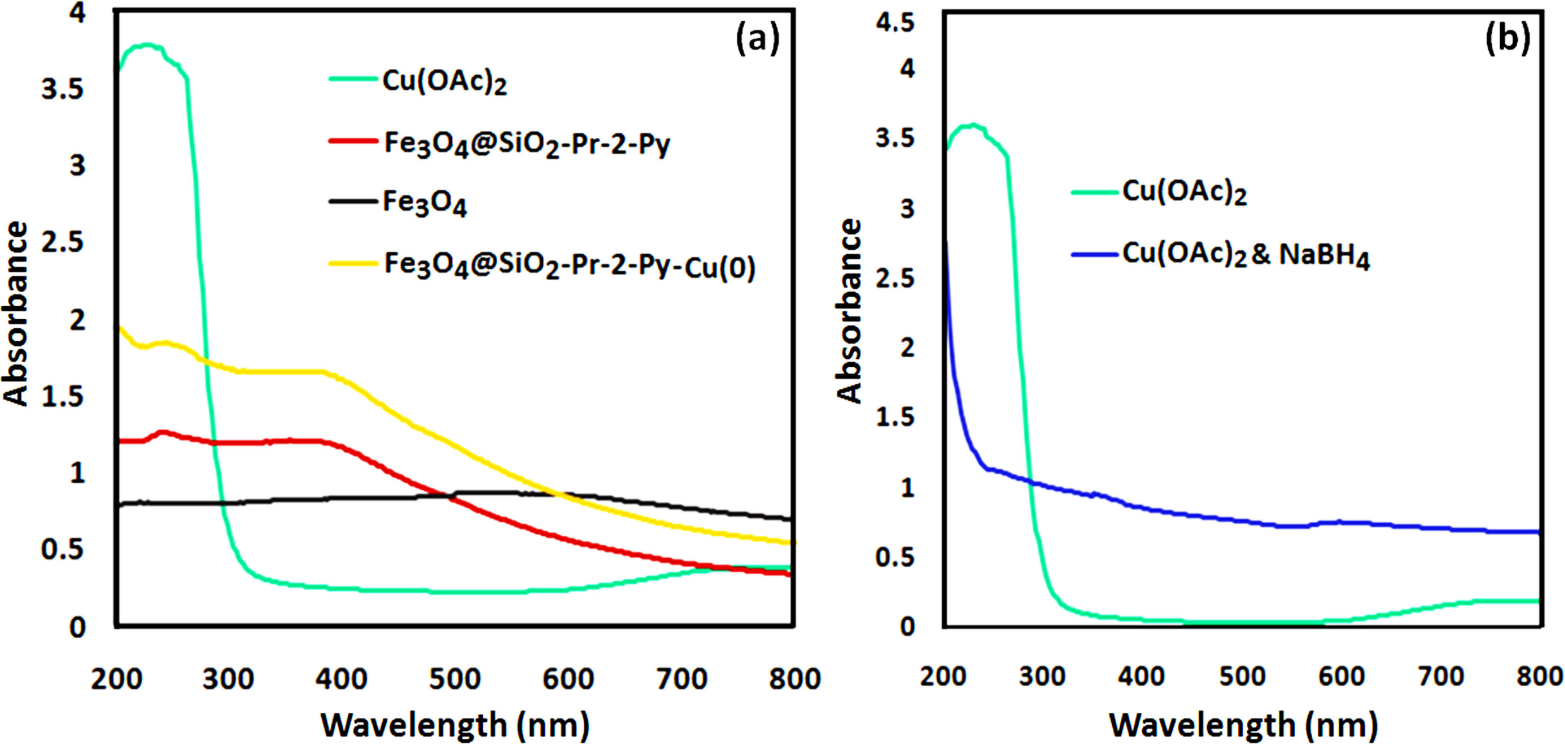
UV-Vis spectra of (*a*) Fe_3_O_4_, Cu(OAc)_2_, Fe_3_O_4_@SiO_2_-Pr-2-Py , and Fe_3_O_4_@SiO_2_-Pr−2-Py-Cu(0), and (*b*) Cu(OAc)_2_, Cu(OAc)_2_ and NaBH_4_.

UV-Vis analysis has been utilized to verify the formation of the nanomagnetic Cu complex and determine the oxidation state of copper in the complex. Figure 7*a* shows the UV-Vis spectra of Cu(OAc)_2_, Fe_3_O_4_@SiO_2_-Pr-2-Py, Fe_3_O_4_ , and Fe_3_O_4_@SiO_2_-Pr-2-Py-Cu(0). The characteristic absorption peak at 270 nm corresponds to the π–π* transition of Cu(II) in Cu(OAc)_2_ [49]. Upon the formation of the complex, the peak corresponding to copper(II) is no longer observed, demonstrating the complete conversion of Cu(II) to Cu(0) in the oxidation state (figure 7*a*). To certify the reduction of Cu(II) to Cu(0) during the last step of the catalyst synthesis, the UV-Vis analysis of copper acetate was carried out in the absence and the presence of sodium borohydride (NaBH_4_) as a reducing agent. These experiments revealed the vanishing of the absorption peak of copper(II) at 270 nm upon the addition of NaBH_4_, which is an indication of a change in the oxidation state of copper from 2 to 0 [50] (figure 7*b*).

The copper content of Fe_3_O_4_@SiO_2_-Pr-2-Py-Cu(0) was determined using the ICP analysis. Based on the results, the Cu loading in Fe_3_O_4_@SiO_2_-Pr-2-Py-Cu(0) nanocatalyst was found to be 0.25 mmol g^−1^. To assess the catalytic activity of this heterogeneous nonmagnetic catalyst in the synthesis of 1,4-disubstituted 1,2,3-triazoles, the one-pot tricomponent reaction between benzyl bromide, phenylacetylene and sodium azide was elected as a prototype reaction to find out the best reaction conditions (table 1). Firstly, the model reaction efficiency was evaluated in diverse solvents (4 ml) using Fe_3_O_4_@SiO_2_-Pr-2-Py-Cu(0) (15 mg, 0.375 mol% Cu) as the catalyst at a temperature of 40°C. The outcomes of these experiments are documented in table 2 (entries 1−9). The data suggest that the reaction gives high performance in protic solvents in comparison to aprotic solvents. The efficiency of the prototype reaction was also tested under solvent-free conditions. However, only a negligible amount of the desired 1,2,3-triazole was obtained after 2 h (table 1, entry 10).

**Table 1.** Optimizing reaction conditions in the preparation of 1-benzyl-4-phenyl triazole as the model compound.
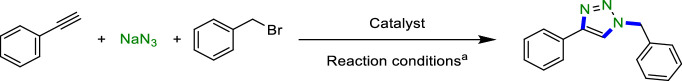

entry	catalyst (mg, mol% Cu)	solvent	time (h)	temp (°C)	yield (%)^b^
1	Fe_3_O_4_@SiO_2_-Pr2-Py-Cu(0) (15 mg, 0.375 mol%)	EtOH	2	40	85
2	Fe_3_O_4_@SiO_2_-Pr-2-Py-Cu(0) (15 mg, 0.375 mol%)	MeOH	2	40	80
3	Fe_3_O_4_@SiO_2_-Pr-2-Py-Cu(0) (15 mg, 0.375 mol%)	H_2_O	2	40	94
4	Fe_3_O_4_@SiO_2_-Pr-2-Py-Cu(0) (15 mg, 0.375 mol%)	CH_2_Cl_2_	2	40	40
5	Fe_3_O_4_@SiO_2_-Pr-2-Py-Cu(0) (15 mg, 0.375 mol%)	THF	2	40	45
6	Fe_3_O_4_@SiO_2_-Pr-2-Py-Cu(0) (15 mg, 0.375 mol%)	DMF	2	40	50
7	Fe_3_O_4_@SiO_2_-Pr-2-Py-Cu(0) (15 mg, 0.375 mol%)	CHCl_3_	2	40	45
8	Fe_3_O_4_@SiO_2_-Pr-2-Py-Cu(0) (15 mg, 0.375 mol%)	DMSO	2	40	55
9	Fe_3_O_4_@SiO_2_-Pr-2-Py-Cu(0) (15 mg, 0.375 mol%)	CH_3_CN	2	40	50
10	Fe_3_O_4_@SiO_2_-Pr-2-Py-Cu(0) (15 mg, 0.375 mol%)	solvent-free	2	40	trace
11	Fe_3_O_4_@SiO_2_-Pr-2-Py-Cu(0) (15 mg, 0.375 mol%)	H_2_O	3	25	40
12	Fe_3_O_4_@SiO_2_-Pr-2-Py-Cu(0) (15 mg, 0.375 mol%)	H_2_O	1	60	95
13	Fe_3_O_4_@SiO_2_-Pr-2-Py-Cu(0) (15 mg, 0.375 mol%)	H_2_O	1	80	95
14	Fe_3_O_4_@SiO_2_-Pr-2-Py-Cu(0) (10 mg, 0.25 mol%)	H_2_O	1	60	97
15	Fe_3_O_4_@SiO_2_-Pr-2-Py-Cu(0) (5 mg, 0.125 mol%)	H_2_O	2	60	80
16	Fe_3_O_4_@SiO_2_-Pr-2-Py-Cu(0) (20 mg, 0.5 mol%)	H_2_O	2	60	94
17	Fe_3_O_4_@SiO_2_-Pr-2-Py-Cu(0) (10 mg, 0.25 mol%)	H_2_O	0.25	60	70
**18**	**Fe_3_O_4_@SiO_2_-Pr-2-Py-Cu(0) (10 mg, 0.25 mol%)**	**H_2_O**	**0.5**	**60**	**97**
19	None	H_2_O	2	60	5
20	Fe_3_O_4_ (20 mg)	H_2_O	3	60	20
21	Fe_3_O_4_@SiO_2_-Pr-2-Py (20 mg)	H_2_O	3	60	25
22	Cu(OAc)_2_ (2 mol%)	H_2_O	5	60	50
23	CuCl_2_(2 mol%)	H_2_O	5	60	45
24	CuBr (2 mol%)	H_2_O	5	60	58
25	CuO (2 mol%)	H_2_O	5	60	55
26	CuI (2 mol%)	H_2_O	5	60	60

^a^ Reaction conditions: Benzyl bromide (1.0 mmol), phenylacetylene (1.2 mmol), NaN_3_ (1.5 mmol), catalyst(appropriate amount), solvent (4.0 mL).

^b^ Isolated yield.

**Table 2.** Employing Fe_3_O_4_@SiO_2_-Pr-2-Py-Cu(0) catalyst in the production of 1,4-disubstituted 1,2,3-triazole derivatives.
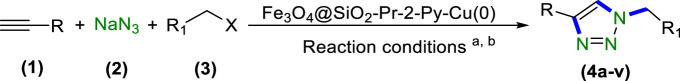

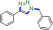	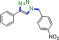	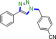
**(4a)^a^** X = Br: 0.5 h, 97%, TOF = 776 (h^−1^) Cl: 0.75 h, 92%, TOF = 490.6 (h^−1^)	**(4b)^a^** X = Br: 0.75 h, 94%, TOF = 501.33 (h^−1^) Cl: 1.5 h, 90%, TOF = 240 (h^−1^)	**(4c)^a^** X = Br: 1 h, 91%, TOF = 364 (h^−1^) Cl: 1.5 h, 88%, TOF = 234.6 (h^−1^)
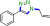	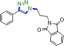	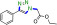
**(4d)^a^** X = Br: 2 h, 88%, TOF = 176 (h^−1^) Cl: 2.5 h, 85%, TOF = 136 (h^−1^)	**(4e)^a^** X = Br: 3.5 h, 85%, TOF = 97.14 (h^−1^) Cl: 4 h, 83%, TOF = 83 (h^−1^)	**(4f)^a^** X = Br: 1.5 h, 89%, TOF = 237.33 (h^−1^) Cl: 2 h, 87%, TOF = 174 (h^−1^)
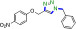	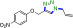	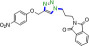
**(4g)^a^** X = Br: 0.5 h, 93%, TOF = 744 (h^−1^) Cl: 1 h, 91%, TOF = 364 (h^−1^)	**(4h)^a^** X = Br: 2.5 h, 86%, TOF = 137.6 (h^−1^) Cl: 3 h, 82%, TOF = 109.3 (h^−1^)	**(4i)^a^** X = Br: 4 h, 82%, TOF = 82 (h^−1^) Cl: 4.75 h, 81%, TOF = 68.2 (h^−1^)
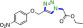	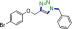	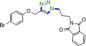
**(4j)^a^** X = Br: 2 h, 87%, TOF = 174 (h^−1^) Cl: 2.5 h, 85%, TOF = 136 (h^−1^)	**(4k)^a^** X = Br: 0.75 h, 92%, TOF = 490.66 (h^−1^) Cl: 1.5 h, 91%, TOF = 242.6 (h^−1^)	**(4l)^a^** X = Br: 3.5 h, 85%, TOF = 97.14 (h^−1^) Cl: 4.25 h, 81%, TOF = 76.2 (h^−1^)
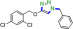	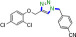	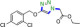
**(4m)^a^** X = Br: 1 h, 94%, TOF = 376 (h^−1^) Cl: 1.25 h, 91%, TOF = 291.2 (h^−1^)	**(4n)^a^** X = Br: 1.5 h, 90%, TOF = 240 (h^−1^) Cl: 2 h, 86%, TOF = 172 (h^−1^)	**(4o)^a^^a^** X = Br: 2 h, 89% TOF = 175 (h^−1^) Cl: 2.25 h, 84%, TOF = 149.3 (h^−1^)
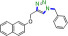	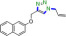	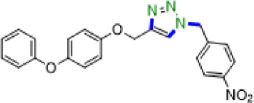
**(4p)^a^** X = Br: 1.5 h, 90%, TOF = 240 (h^−1^) Cl: 1.75 h, 85%, TOF = 194.2 (h^−1^)	**(4q)^a^** X = Br: 2.5 h, 84%, TOF = 134.4 (h^−1^) Cl: 3 h, 82%, TOF = 109.3 (h^−1^)	**(4r)^a^** X = Br: 1.5 h, 90%, TOF = 240 (h^−1^) Cl: 2 h, 89%, TOF = 178 (h^−1^)
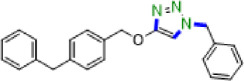		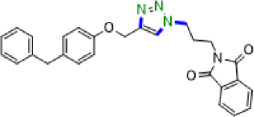
**(4s)^a^** X = Br: 1.25 h, 93%, TOF = 297.6 (h^−1^) Cl: 1.75 h, 91%, TOF = 208 (h^−1^)		**(4t)^a^** X = Br: 3.5 h, 84 %, TOF = 96 (h^−1^) Cl: 4 h, 80%, TOF = 80 (h^−1^)
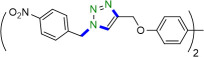		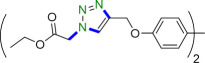
**(4u)^b^** X = Br: 4.5 h, 84%, TOF = 74.66 (h^−1^) Cl: 5.25 h, 81%, TOF = 30.8 (h^−1^)		**(4v)^b^** X = Br: 5 h, 81%, TOF = 64.8 (h^−1^) Cl: 6 h, 80%, TOF = 26.6 (h^−1^)

^a^ Reaction conditions: NaN_3_ (1.5 mmol), Alkyne (1.2 mmol), organic halide (1 mmol), Fe_3_O_4_@SiO_2_-Pr-2-Py-Cu(0) (0.25 mol% Cu) in H_2_O(4 mL) at 60°C.

^b^ Reaction conditions: NaN_3_ (3 mmol), Alkyne (1.2 mmol), organic halide (2 mmol), Fe_3_O_4_@SiO_2_-Pr-2-Py-Cu(0) (0.5 mol%Cu) in H_2_O (6 mL) at 60°C; yields refer to isolated yields.

The reaction was also conducted at various temperatures (25, 60 and 80°C). When the reaction temperature increased from ambient temperature to 60°C, the efficiency enhanced from 40 to 95%, but no improvement was detected in the efficiency through raising the temperature to 80°C (table 1, entries 11−13). The optimization study revealed that consuming 10 mg of the magnetic nanocatalyst, which is equivalent to 0.25 mol% Cu, offers the best catalytic role at 60°C in H_2_O after 1 h (table 1, entry 14). Loading a lower dose of the catalyst, 0.125 mol% Cu, led to a lower yield of the title product (table 1, entry 15), and applying a higher dose of the catalyst did not exhibit any improvement in the reaction efficiency even after 2 h (table 1, entry 16). Running the reaction using 10 mg of the catalyst at 600°C in H_2_O in a shorter reaction time than 0.5 h resulted in a lower yield of the desired product (table 1, entry 17), and performing the reaction in a longer time, 1 h, did not enhance the reaction efficiency (table 1, entries 14 and 18).

To assess the catalyst’s performance and its pivotal role, the prototype reaction was carried out in the absence of the catalyst and the presence of Fe_3_O_4_ and Fe_3_O_4_@SiO_2_-Pr-2-Py separately, which were terminated to 5, 20 and 25% yield of the expected product, respectively (table 1, entries 19−21). The catalytic activity of various copper sources, including CuBr, CuI, Cu(OAc)_2_, CuO and CuCl_2_, was also checked in the reaction involving sodium azide, benzyl bromide and phenylacetylene in aqueous medium at a temperature of 60°C. The outcomes of these experiments demonstrated a moderate yield of the desired triazole compound (table 1, entries 22−26). Based on the data presented in table 1, the subsequent phases of the study were carried out employing water as an eco-friendly solvent alongside Fe_3_O_4_@SiO_2_-Pr-2-Py-Cu(0) (10 mg) under optimized conditions at 60°C.

Once the optimal reaction conditions were determined, further investigation was conducted to explore the generality and scope of the developed strategy in synthesizing 1,4-disubstituted 1,2,3-triazoles. Various organic halide and alkyne compounds were used as starting substrates to produce the corresponding triazoles (table 2). The results showed that brominated organic compounds exhibited higher efficiency and shorter reaction times compared with chlorinated organic compounds (table 2) [5]. Among benzyl halides with electron-withdrawing substituents such as NO_2_ and CN, the reaction took longer with slightly lower yields than benzyl bromide itself (table 2) [4,5] . Halides such as allyl bromide, ethyl bromoacetate and *N*-(3-bromopropyl) phthalimide performed well in the triazole formation reaction but provided lower product yields in longer reaction times than benzyl bromides (table 2, entries 4d-4f).

**Scheme 3.**
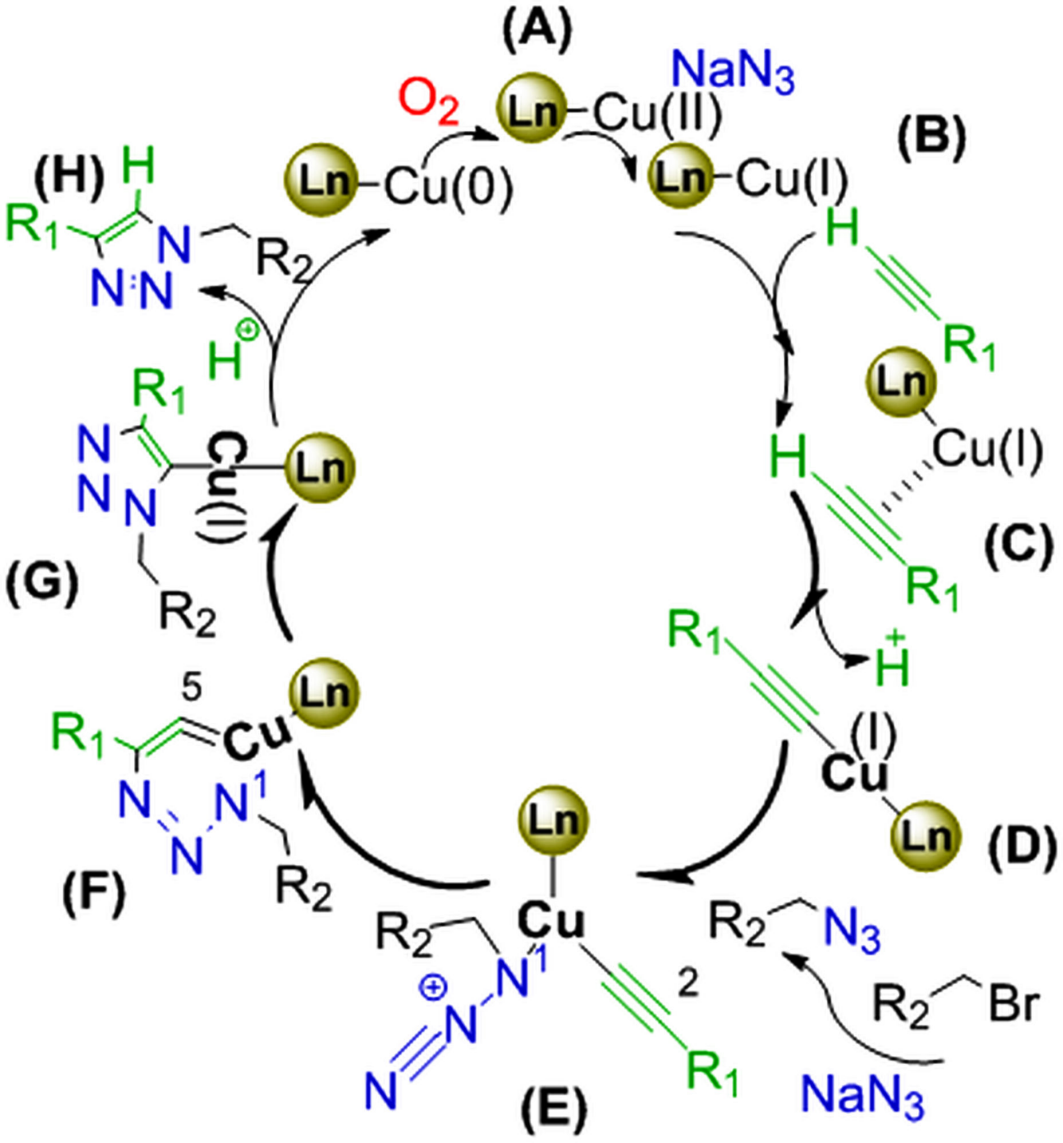
Plausible mechanism for the formation of 1,4-disubstituted 1,2,3-triazoles.

In order to investigate the possible events that may occur during the formation of 1,4-disubstituted 1,2,3-triazoles, a tentative mechanism has been proposed (scheme 3) [51]. Initially, the Cu(0) nanoparticles are oxidized to the Cu(II) species (**A**)[52]**,** which is reduced in turn to the Cu(I) nanoparticles (**B**) with sodium azide as one of the required starting materials to provide **B** [23,53]. The formation of the π complex alkyne-Cu(I) **C** result in the displacement of H^+^ with Cu(I) and the generation of σ-bond copper(I) acetylide **D**. As a matter of fact, the formation of Cu(I) with a terminal alkyne decreases the p*K*a of the acetylenic proton up to 9.8, enabling deprotonation in the aqueous system without the need for a strong base [54,55]. Then the activation azide by the *in situ* generated organic azide via coordinating to the Cu centre and nucleophilic attack of the nitrogen azide on the σ-complex **D** leads to the formation of intermediate **E**, which undergoes an intermolecular cyclization through attacking of the terminal nitrogen atom of the azide group on C2 to provide the transient molecule **F**. This six-membered metallocyclic compound **F** carries out N1 transfer to C5 to provide the substituted 1,2,3-triazolyl organocopper(I) G, which eventually is converted to the related 1,4-disubstituted 1,2,3-triazole **H** and regenerates the Cu(0) nanomagnetic catalyst after undergoing protonolysis reaction.

The catalytic activity of the present nanocatalyst in comparison to the copper catalysts that have recently been reported in the chemistry literature for the preparation of 1-benzyl-4-phenyl triazole from the related starting material as the standard reaction is shown in table 3. As is shown in table 3, the Fe_3_O_4_@SiO_2_-Pr-2-Py-Cu(0) MNPs provide greener reaction conditions with higher TOFs (table 3, entry 3) while the other copper catalysts act either in harsh conditions or with lower efficiencies (table 3, entries 1 and 2).

**Table 3.** Efficiency comparison of Fe_3_O_4_@SiO_2_-Pr-2-Py-Cu(0) nanocatalyst with several recently published copper catalysts in the formation of 1-benzyl-4-phenyl triazole.

entry	catalyst (mol% Cu)	reaction conditions ^a^	time (h)	yield (%)	TOF (h^-1^)	reference
1	Fe_3_O_4_@HKUST−1 (1.8 mol%)	H_2_O, 90°C	3	92	17	[56]
2	GO-SB-Cu (1.6 mol%)	MeOH, rt	4	91	14	[57]
**3**	**Fe_3_O_4_@SiO_2_-Pr-2-Py-Cu(0) (0.25 mol%)**	**H_2_O, 60°C**	**0.5**	**97**	**776**	**this work**

^a^ Reaction conditions: Alkyne (1.2 mmol), benzyl bromide (1 mmol), NaN_3_ (1.5 mmol).

The efficient catalytic reactivity of Fe_3_O_4_@SiO_2_-Pr-2-Py-Cu(0) in the synthesis of 1,4-disubstituted 1,2,3-triazoles prompted its examination in the one-pot synthesis of 5-substituted 1*H*-tetrazole. Initially, a standard reaction involving benzaldehyde, sodium azide and hydroxylamine HCl was picked out for the optimization of reaction conditions (table 4). We optimized several parameters including the solvent, time, catalyst amount and temperature. Oxime consumption was monitored in all reactions using TLC because oxime formation occurs faster than its reaction with azide to produce tetrazole in the proposed method [29]. In order to examine the impact of temperature, we conducted the mentioned model reaction using 20 mg of Fe_3_O_4_@SiO_2_-Pr-2-Py-Cu(0) (0.5 mol% Cu) and 5 ml H_2_O at room temperature. The oxime formed rapidly, but the tetrazole did not (table 4, entry 1). The reaction was also conducted at 50 and 75°C, and under reflux conditions, showing an increase in the product formation with rising temperature (table 4, entries 2−4). The best result was obtained under reflux conditions. Subsequently, different catalyst amounts were tested, revealing that increasing the catalyst amount up to 16 mg (0.4 mol% Cu) improved the reaction efficiency while using a higher amount of 16 mg did not result in any further improvement (table 4, entries 5 and 6). The influence of various solvents on the model reaction was also assessed by conducting the experiment using various solvents (table 4, entries 7−12). The choice of solvent had a significant impact on both catalyst activity and product yield. Moderate yields were obtained when the reaction was conducted in MeOH, THF, EtOH and CH_3_CN under reflux conditions for 6 h (table 4, entries 7−10), while the solvent-free condition was not suitable for this reaction (table 4, entry 13). Although the reaction proceeded with excellent yield in DMSO at 120°C and DMF at 110°C (table 4, entries 11 and 12), the highest efficiency and reactivity were achieved in boiling H_2_O after 3 h (table 4, entry 6). Therefore, water, being the most economical, safest and environmentally friendly solvent, was selected as the best solvent for further study on the scope and versatility of this reaction. To enhance the response time, we evaluated the reaction at various durations and determined that the optimal time is 2 h (table 4, entries 14 and 15), resulting in a 95% yield under pre-optimized conditions (table 4, entry 15). When the reaction was performed in the absence of the catalyst, a trace amount of the product was obtained after 6 h (table 4 , entry 16). The reaction was examined in the existence of bare Fe_3_O_4_ and Fe_3_O_4_@SiO_2_-Pr-2-Py and led to a trace amount and 10% yield of the desired tetrazole, respectively (table 4, entries 17 and 18). Moderate yields were also achieved when Cu(OAc)_2_, CuI, Cu_2_O and CuCl_2_ were used as the copper sources (table 4 , entries 19−22).

**Table 4.** Optimizing reaction parameters in the formation of 5-phenyltetrazole.
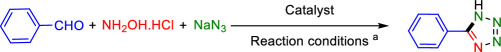

entry	catalyst (mg, mol% Cu)	solvent	time (h)	temp. (℃)	yield (%)^b^
1	Fe_3_O_4_@SiO_2_-Pr-2-Py-Cu(0) (20 mg, 0.5 mol%)	H_2_O	6	25	trace
2	Fe_3_O_4_@SiO_2_-Pr-2-Py-Cu(0) (20 mg, 0.5 mol%)	H_2_O	5	50	50
3	Fe_3_O_4_@SiO_2_-Pr-2-Py-Cu(0) (20 mg, 0.5 mol%)	H_2_O	5	75	75
4	Fe_3_O_4_@SiO_2_-Pr-2-Py-Cu(0) (20 mg, 0.5 mol%)	H_2_O	3	reflux	92
5	Fe_3_O_4_@SiO_2_-Pr-2-Py-Cu(0) (10 mg, 0.25 mol%)	H_2_O	5	reflux	80
6	Fe_3_O_4_@SiO_2_-Pr-2-Py-Cu(0) (16 mg, 0.4 mol%)	H_2_O	r3	reflux	94
7	Fe_3_O_4_@SiO_2_-Pr-2-Py-Cu(0) (16 mg, 0.4 mol%)	THF	6	reflux	40
8	Fe_3_O_4_@SiO_2_-Pr-2-Py-Cu(0) (16 mg, 0.4 mol%)	MeOH	6	reflux	50
9	Fe_3_O_4_@SiO_2_-Pr-2-Py-Cu(0) (16 mg, 0.4 mol%)	EtOH	6	reflux	70
10	Fe_3_O_4_@SiO_2_-Pr-2-Py-Cu(0) (16 mg, 0.4 mol%)	CH_3_CN	6	reflux	40
11	Fe_3_O_4_@SiO_2_-Pr-2-Py-Cu(0) (16 mg, 0.4 mol%)	DMSO	6	120	90
12	Fe_3_O_4_@SiO_2_-Pr-2-Py-Cu(0) (16 mg, 0.4 mol%)	DMF	6	110	92
13	Fe_3_O_4_@SiO_2_-Pr-2-Py-Cu(0) (16 mg, 0.4 mol%)	solvent-free	6	100	Trace
14	Fe_3_O_4_@SiO_2_-Pr-2-Py-Cu(0) (16 mg, 0.4 mol%)	H_2_O	1	reflux	70
**15**	**Fe_3_O_4_@SiO_2_-Pr-2-Py-Cu(0) (16 mg, 0.4 mol%)**	**H_2_O**	**2**	**reflux**	**95**
16	None	H_2_O	6	reflux	trace
17	Fe_3_O_4_ (16 mg)	H_2_O	6	reflux	trace
18	Fe_3_O_4_@SiO_2_-Pr-2-Py (16 mg)	H_2_O	6	reflux	10
19	Cu(OAc)_2_(5 mol%)	H_2_O	8	reflux	30
20	CuCl_2_(5 mol%)	H_2_O	8	reflux	35
21	Cu_2_O (5 mol%)	H_2_O	8	reflux	25
22	CuI (5 mol%)	H_2_O	8	reflux	20

^a^ Reaction conditions: Benzaldehyde (1 mmol), hydroxylamine hydrochloride (1.2 mmol), NaN_3_ (1.5 mmol), catalyst (appropriate amount) and solvent (5 ml).

^b^ Isolated yield.

**Table 5.** Fe_3_O_4_@SiO_2_-Pr-2-Py-Cu(0) catalysed formation of 5-substituted 1*H*-tetrazoles.
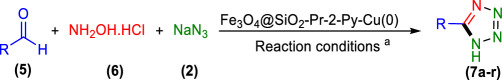

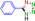	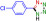	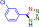
**(7a)** 2 h, 95%,^b^ TOF = 118.75 h^−1^	**(7b)** 1.5 h, 94%,^b^ TOF = 156.66 h^−1^	**(7c)** 2.5 h, 87%,^b^ TOF = 87 h^−1^
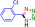	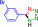	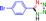
**(7d)** 3 h, 84%,^b^ TOF = 70 h^−1^	**(7e)** 3 h, 88%,^b^ TOF = 73.33 h^−1^	**(7f)** 2 h, 96%,^b^ TOF = 120 h^−1^
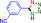	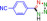	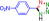
**(7g)** 2.5 h, 90%,^b^ TOF = 90 h^−1^	**(7h)** 2 h, 92%,^b^ TOF = 115 h^−1^	**(7i)** 1.5 h, 94%,^b^ TOF = 156.66 h^−1^
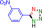		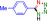
**(7j)** 2.25 h, 91%,^b^ TOF = 101.11 h^−1^	**(7k)** 3 h, 84%,^b^ TOF = 70 h^−1^	**(7l)** 4 h, 90%,^b^ TOF = 56.25 h^−1^
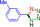	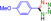	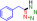 −
**(7m)** 4.5 h, 88 %,^b^ TOF = 48.88 h^−1^	**(7n)** 3.5 h, 92%,^b^ TOF = 65.71 h^−1^	**(7o)** 6 h, 82%,^b^ TOF = 34.1 h^−1^7 h
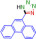	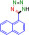	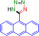
**(7p)** 8 h, 82%,^b^ TOF = 25.62 h^−1^	**(7q)** 3.25 h, 89%,^b^ TOF = 68.4 h^−1^	**(7r)** 7 h, 84 %,^b^ TOF = 30 h^−1^

^a^ Reaction conditions: Aldehyde (1 mmol), NaN_3_ (1.5 mmol), hydroxylamine hydrochloride (1.2 mmol),Fe_3_O_4_@SiO_2_-Pr-2-Py-Cu(0) (0.4 mol% Cu) and H_2_O (5 mL) at reflux condition.

^b^ Isolated yield.

After maximizing the reaction parameters, the potential of the protocol was evaluated in the formation of 5-substituted 1*H*-tetrazole derivatives using various aromatic aldehydes. As demonstrated in table 5, aldehydes bearing electron-withdrawing groups exhibited slightly greater efficiency in comparison to those with electron-donating substituents. This method, in general, does not show significant sensitivity to the electronic nature of the substituents. The steric hindrance of the substrate has a notable impact on the yield and reaction time. So aromatic aldehydes with a substituent at the *ortho* position underwent this reaction at longer times and with lower yields in comparison to aromatic aldehydes with the same substituent at the *meta* and *para* positions. Hindered polynuclear aromatic aldehydes, such as 9-phenanthrenecarboxaldehyde and 9-anthracenecarboxaldehyde, afforded the tetrazole formation in 82 and 84% yield in 8 and 7 h, respectively (table 5, entries 7p and 7r). Additionally, 2-phenylacetaldehyde, an aliphatic aldehyde, was successfully transformed into the corresponding tetrazole with a yield of 82% in 6 h (table 5, entry 7o).

**Scheme 4.**
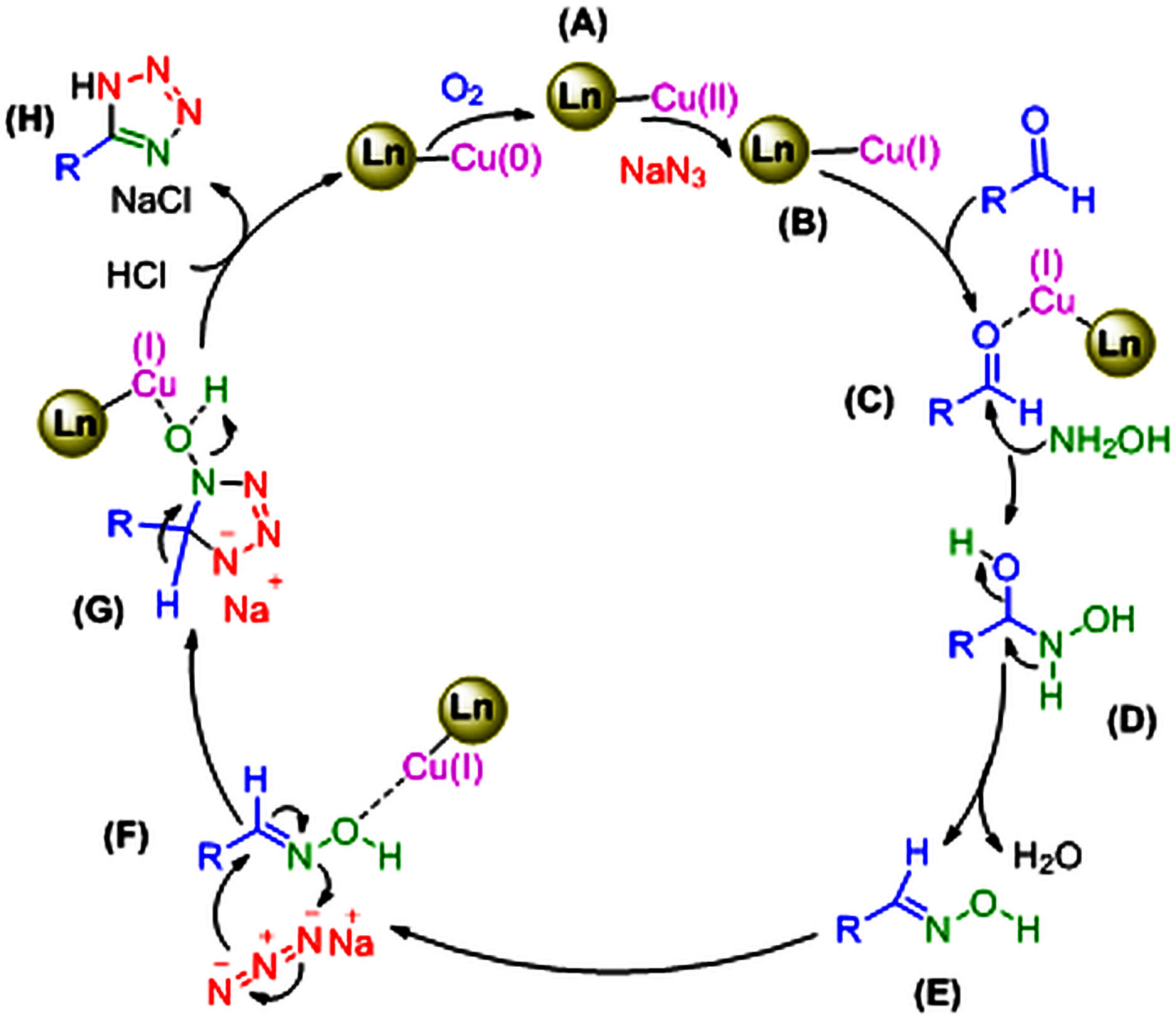
Plausible mechanism for the formation of tetrazoles.

According to the literature study, two general paths for the formation of 5-substituted 1*H*-tetrazole compounds have been reported from oximes. In the first path, aldoxime is converted to the related nitrile through water loss and then reacts with NaN_3_ to form a tetrazole. In the second path, aldoxime is converted directly to tetrazole via a reaction with NaN_3_. So to find out how Fe_3_O_4_@SiO_2_-Pr-2-Py-Cu(0) might work, three experiments were conducted. In the first experiment, benzaldehyde was treated with hydroxylamine HCl in the presence of nanocatalyst-Fe_3_O_4_@SiO_2_-Pr-2-Py-Cu(0) in water, which instantly led to benzaldoxime at ambient temperature. In the second experiment, for checking the conversion of aldoxime to the related nitrile, benzaldoxime was heated in boiling water in the presence of the catalyst for 2 h, but it did not generate benzonitrile. In the third experiment, the reaction between aldoxime and sodium azide occurred after 2 h, leading to the formation of 5-phenyl-1*H*-tetrazole. Based on these results, the mechanism illustrated in scheme 4 suggests that Cu(0) is initially oxidized to Cu(II) in the presence of oxygen in the air (**A**)^52^. Then, the reduction of Cu(II) to Cu(I) by sodium azide (**B**) will take place, as it is one of the necessary starting materials to obtain the complex (**C**) [23, 25, 58]. This complex undergoes a nucleophilic addition by hydroxylamine (**D**) to provide the oxime (**E**) after losing water. The activated oxime F by the Cu(I)-catalyst undergoes [3+2] cycloaddition reaction with NaN_3_ to produce anion species (**G),** which is converted to the related tetrazole (**H)** and regenerates the Cu(0) catalyst when treated with hydrochloric acid.

Assessing the capabilities of the prepared magnetic nanocatalyst in comparison with several recent reports in the preparation of 5-(4-chlorophenyl)-1*H*-tetrazole revealed that Fe_3_O_4_@SiO_2_-Pr-2-Py-Cu(0) works more efficiently in water as a greener environment under milder reaction conditions, leading to a notably better TOF value (table 6).

**Table 6.** Comparison of Fe_3_O_4_@SiO_2_-Pr-2-Py-Cu(0) in the synthesis of 5-(4-chlorophenyl)-1*H*-tetrazole with several recently reported Cu catalysts.

entry	catalyst (mol% Cu)	reaction conditions ^a^	time (h)	yield (%)	TOF (h^−1^)	reference
1	Fe_3_O_4_@MCM-41-SB-Cu (3.48 mol%)	DMF, 120°C	1.5	88	16.8	[59]
2	Copper(II) Schiff base (0.5 mol%)	DMF, 110°C	7	93	26.5	[60]
3	**Fe_3_O_4_@SiO_2_-Pr-2-Py-Cu(0) (0.4 mol%)**	**H_2_O, reflux**	**1.5**	**94**	**156.6**	**this work**

^a^ Reaction conditions: Benzaldehyde (1 mmol), NaN_3_ (1.5 mmol), hydroxylamine hydrochloride (1.2 mmol).

**Figure 8.**
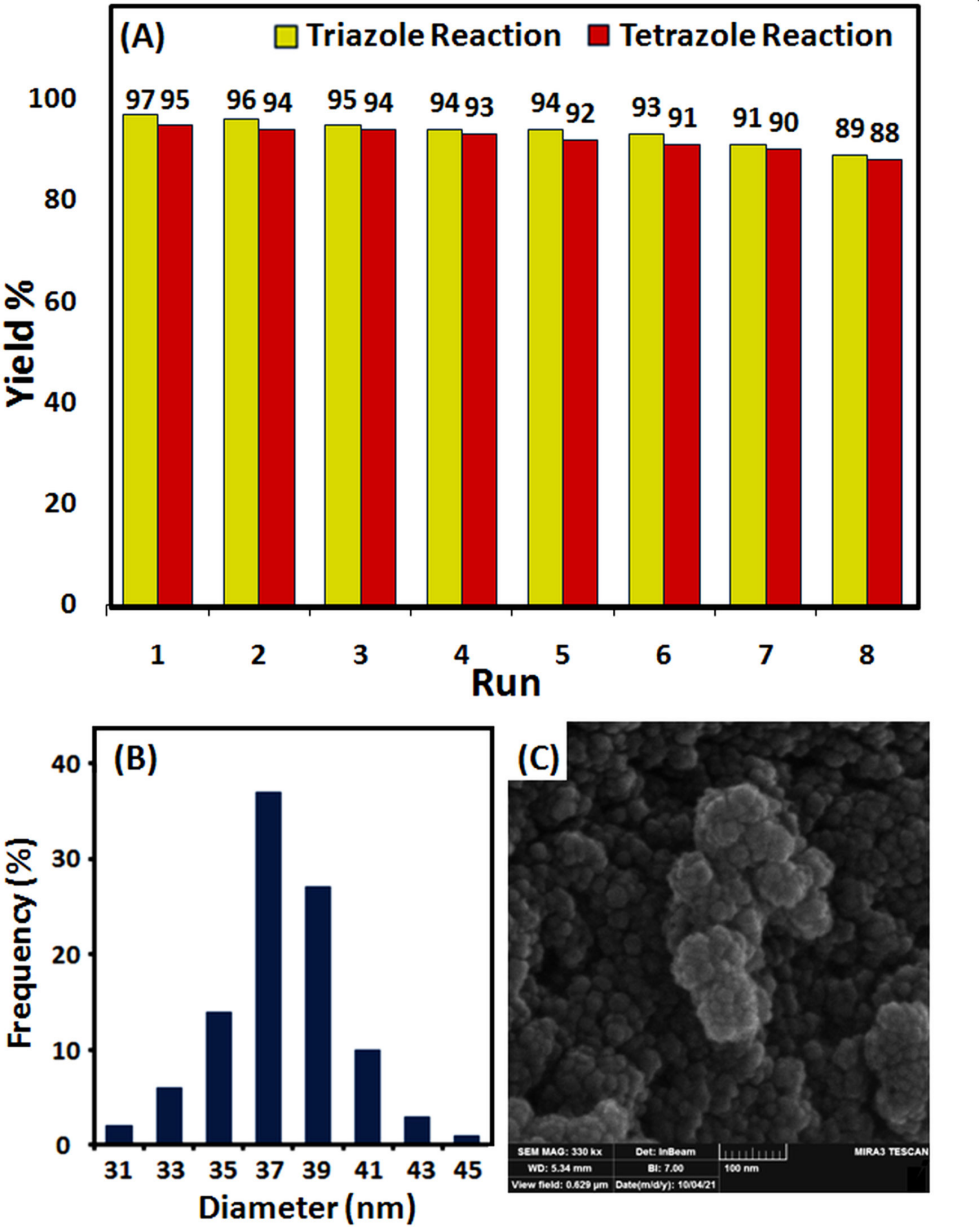
(*a*) Recyclability of Fe_3_O_4_@SiO_2_-Pr-2-Py-Cu(0) in the synthesis of 1-benzyl-4-phenyl-1*H*-1,2,3-triazole and 5-phenyl-1*H*-tetrazole; (*b*) DLS and (*c*) FE-SEM image of Fe_3_O_4_@SiO_2_-Pr-2-Py after eight reaction cycles.

In order to develop a green catalytic process, two essential properties are the restorability and reusability of the catalyst. Accordingly, these criteria were explored in the synthesis of 1-benzyl-4-phenyl-1,2,3-triazole and 5-phenyl-1*H*-tetrazole (figure 8*a*). After the reaction, the nanocatalyst, due to its magnetic nature, was readily removed using a magnet. To be reused in the next cycle, it was washed twice with ethyl acetate and then with a mixture of distilled water and ethanol and dried afterwards. This study demonstrated that the copper(0) catalyst could be reused for eight sequential runs without a significant decrease in activation. After the eighth recovery, the product yield in the synthesis of the corresponding triazole and tetrazole declined from 97 and 95% to 89 and 88%, independently (figure 8*a*). One of the most challenging issues in heterogeneous catalysis is the leaching of metal from the catalyst. Therefore, we investigated copper leaching using ICP analysis. The analysis showed that 4.4 and 6.25% of the copper in the catalyst leached out into the solution of the triazole and tetrazole model reactions after eight runs, individually. This small amount of leaching demonstrates the reasonable stability of nanocatalysts in terms of catalytic activation after eight runs.

The FE-SEM analysis of the recovered nanomagnetic catalyst denotes preserved spherical morphology, and according to the DLS histogram, the average particle size is 37 nm, slightly larger than that of the fresh catalyst. These changes in size and dispersion relative to the fresh catalyst are reasonable (figure 8*b*,*c*).

## 4. Conclusion

In conclusion, we developed a novel and magnetically recoverable nanocatalyst, Fe_3_O_4_@SiO_2_-Pr-2-Py-Cu(0), which is environmentally benign and based on Cu(0). The nanocatalyst was synthesized through a green and low-cost multistep process. It was fully characterized through XRD, FT-IR, BET, TGA, FE-SEM, VSM, EDX, ICP, mapping and UV-Vis analysis techniques. The nanocatalyst exhibited exceptional catalytic efficiency in the three-component one-pot eco-friendly preparation of 1,4-disubstituted 1,2,3-triazole and 5-substituted 1*H*-tetrazoles in water, using a small amount of copper content. Compared to the strategies introduced in the literature, this method has several important advantages: working in water as a green and eco-friendly environment under mild reaction conditions at a relatively short reaction time, utilizing an easily magnetically recoverable and eight times reusable catalyst, low catalyst consumption, avoiding the application of external ligands and additives and providing outstanding yields of the desired products.

## Data Availability

The datasets supporting this article have been uploaded as part of the supplementary material [61].

## References

[1] Khanmohammadi‐Sarabi F, Ghorbani‐Choghamarani A, Aghavandi H, Zolfigol MA. 2022 ZnFe_2_O_4_@SiO_2_-ascorbic acid: Green, magnetic, and versatile catalyst for the synthesis of chromeno[2,3-d] pyrimidine-8-amine and quinazoline derivatives. Appl. Organomet. Chem **36**, 6768. (10.1002/aoc.6768)

[2] Gawande MB, Luque R, Zboril R. 2014 The Rise of Magnetically Recyclable Nanocatalysts. ChemCatChem **6**, 3312–3313. (10.1002/cctc.201402663)

[3] Lei Z, Li Y, Wei X. 2008 A facile two-step modifying process for preparation of poly(SStNa)-grafted Fe_3_O_4_/SiO_2_ particles. J. Solid State Chem. **181**, 480–486. (10.1016/j.jssc.2007.12.004)

[4] Bagherzadeh N, Sardarian AR, Eslahi H. 2021 Sustainable and recyclable magnetic nanocatalyst of 1,10-phenanthroline Pd(0) complex in green synthesis of biaryls and tetrazoles using arylboronic acids as versatile substrates. Mol. Catal. **504**, 111489. (10.1016/j.mcat.2021.111489)

[5] Bagherzadeh N, Reza Sardarian A. 2024 Green approach to new library of 1,8-naphthalimide fluorophores employing C-C and C-N cross coupling reactions by novel, durable, and reusable magnetic nanocatalyst bearing nickel (0) complex of 1,10-phenanthroline. J. Photochem. Photobiol. **449**, 115345. (10.1016/j.jphotochem.2023.115345)

[6] Alonso F, Moglie Y, Radivoy G. 2015 Copper Nanoparticles in Click Chemistry. Accounts Chem. Res. **48**, 2516–2528. (10.1021/acs.accounts.5b00293) 26332570

[7] Wang Y, Biradar AV, Wang G, Sharma KK, Duncan CT, Rangan S, Asefa T. 2010 Controlled Synthesis of Water‐Dispersible Faceted Crystalline Copper Nanoparticles and Their Catalytic Properties. Chem. – Eur. J. **16**, 10735–10743. (10.1002/chem.201000354) 20648484

[8] Rohilla S, Patel SS, Jain N. 2016 Copper Acetate Catalyzed Regio­selective Synthesis of Substituted 1,2,3‐Triazoles: A Versatile Azide–Alk­ene Cycloaddition/Oxidation Approach. Eur. J. Org. Chem. **2016**, 847–854. (10.1002/ejoc.201501301)

[9] Vala DP *et al*. 2024 Click-chemistry mediated synthesis of OTBN-1,2,3-Triazole derivatives exhibiting STK33 inhibition with diverse anti-cancer activities. Bioorganic Chem. **149**, 107485. (10.1016/j.bioorg.2024.107485) 38824700

[10] Upadhyay DB *et al*. 2024 Indole clubbed 2,4‐thiazolidinedione linked 1,2,3‐triazole as a potent antimalarial and antibacterial agent against drug‐resistant strain and molecular modeling studies. Arch. Der Pharm. **357**, e2300673. (10.1002/ardp.202300673) 38247229

[11] Duan T, Fan K, Fu Y, Zhong C, Chen X, Peng T, Qin J. 2012 Triphenylamine-based organic dyes containing a 1,2,3-triazole bridge for dye-sensitized solar cells via a ‘Click’ reaction. Dye. Pigment. **94**, 28–33. (10.1016/j.dyepig.2011.11.008)

[12] Debia NP, Saraiva MT, Martins BS, Beal R, Gonçalves PFB, Rodembusch FS, Alves D, Lüdtke DS. 2018 Synthesis of Amino Acid-Derived 1,2,3-Triazoles: Development of a Nontrivial Fluorescent Sensor in Solution for the Enantioselective Sensing of a Carbohydrate and Bovine Serum Albumin Interaction. J. Org. Chem. **83**, 1348–1357. (10.1021/acs.joc.7b02852) 29313350

[13] Ghosh D *et al*. 2015 A simple and effective 1,2,3-triazole based “turn-on” fluorescence sensor for the detection of anions. New J. Chem. **39**, 295–303. (10.1039/c4nj01411a)

[14] Meisner QJ, Accardo JV, Hu G, Clark RJ, Jiang D en, Zhu L. 2018 Fluorescence of Hydroxyphenyl-Substituted “Click” Triazoles. J. Phys. Chem. **122**, 2956–2973. (10.1021/acs.jpca.8b00577) 29489363

[15] Sinopoli A, Black FA, Wood CJ, Gibson EA, Elliott PIP. 2017 Investigation of a new bis(carboxylate)triazole-based anchoring ligand for dye solar cell chromophore complexes. Dalton Trans. **46**, 1520–1530. (10.1039/c6dt02905a) 28091637

[16] Rostovtsev VV, Green LG, Fokin VV, Sharpless KB. 2002 A Stepwise Huisgen Cycloaddition Process: Copper(I)-Catalyzed Regioselective “Ligation” of Azides and Terminal Alkynes. Angew. Chem. Int. Ed. **41**, 2-4 2596–2599 1521-3773. (10.1002/1521-3773(20020715)41:143.0.co;2-4) 12203546

[17] Tornøe CW, Christensen C, Meldal M. 2002 Peptidotriazoles on Solid Phase: [1,2,3]-Triazoles by Regiospecific Copper(I)-Catalyzed 1,3-Dipolar Cycloadditions of Terminal Alkynes to Azides. J. Org. Chem. **67**, 3057–3064. (10.1021/jo011148j) 11975567

[18] Liang L, Astruc D. 2011 The copper(I)-catalyzed alkyne-azide cycloaddition (CuAAC) “click” reaction and its applications. An overview. Coord. Chem. Rev. **255**, 2933–2945. (10.1016/j.ccr.2011.06.028)

[19] Wei F, Wang W, Ma Y, Tung CH, Xu Z. 2016 Regioselective synthesis of multisubstituted 1,2,3-triazoles: moving beyond the copper-catalyzed azide–alkyne cycloaddition. Chem. Commun. **52**, 14188–14199. (10.1039/c6cc06194j) 27711308

[20] Alexander JR, Ott AA, Liu EC, Topczewski JJ. 2019 Kinetic Resolution of Cyclic Secondary Azides, Using an Enantioselective Copper-Catalyzed Azide–Alkyne Cycloaddition. Org. Lett. **21**, 4355–4358. (10.1021/acs.orglett.9b01556) 31117717 PMC6631410

[21] Michinobu T, Diederich F. 2018 The [2+2] Cycloaddition‐Retroelectrocyclization (CA‐RE) Click Reaction: Facile Access to Molecular and Polymeric Push‐Pull Chromophores. Angew. Chem. Int. Ed. **57**, 3552–3577. (10.1002/anie.201711605) 29469183

[22] Lal S, Díez-González S. 2011 [CuBr(PPh_3_)_3_] for Azide−Alkyne Cycloaddition Reactions under Strict Click Conditions. J. Org. Chem **76**, 2367–2373. (10.1021/jo200085j) 21384852

[23] Bagherzadeh N, Amiri M, Sardarian AR. 2023 Novel Cu(ii) acidic deep eutectic solvent as an efficient and green multifunctional catalytic solvent system in base-free conditions to synthesize 1,4-disubstituted 1,2,3-triazoles. RSC Adv. **13**, 36403–36415. (10.1039/d3ra06570g) 38099257 PMC10719904

[24] Castillo JC, Bravo NF, Tamayo LV, Mestizo PD, Hurtado J, Macías M, Portilla J. 2020 Water-Compatible Synthesis of 1,2,3-Triazoles under Ultrasonic Conditions by a Cu(I) Complex-Mediated Click Reaction. ACS Omega **5**, 0c04592. (10.1021/acsomega.0c04592) PMC7689893 33251449

[25] Nuñez-Dallos N, Muñoz-Castro A, Fuentealba M, Pérez EG, Hurtado JJ. 2019 Facile synthesis of a luminescent copper(I) coordination polymer containing a flexible benzotriazole-based ligand: An effective catalyst for three-component azide-alkyne cycloaddition. Inorganica Chim. Acta **498**, 119136. (10.1016/j.ica.2019.119136)

[26] Lu J, Ma EQ, Liu YH, Li YM, Mo LP, Zhang ZH. 2015 One-pot three-component synthesis of 1,2,3-triazoles using magnetic NiFe_2_O_4_–glutamate–Cu as an efficient heterogeneous catalyst in water. RSC Adv. **5**, 59167–59185. (10.1039/c5ra09517d)

[27] Emami M, Bikas R, Noshiranzadeh N, Kozakiewicz A, Lis T. 2020 Cu(II)-Hydrazide Coordination Compound Supported on Silica Gel as an Efficient and Recyclable Heterogeneous Catalyst for Green Click Synthesis of β-Hydroxy-1,2,3-triazoles in Water. ACS Omega **5**, 0c01491. (10.1021/acsomega.0c01491) PMC7288712 32548521

[28] Zhang SG, Liang CG, Zhang WH. 2018 Recent Advances in Indazole-Containing Derivatives: Synthesis and Biological Perspectives. Molecules **23**, 2783. (10.3390/molecules23112783) 30373212 PMC6278422

[29] Longbottom D, Franckevicius V, Kumarn S, Oelke A, Wascholowski V, Ley S. 2008 Practical organocatalysis with (S)-and (R)-5-pyrrolidin-2-yl-1H-tetrazoles. Aldrichimica Acta **41**, 3–11.

[30] Klapötke TM, Witkowski TG. 2015 Nitrogen‐Rich Energetic 1,2,5‐Oxadiazole‐Tetrazole – Based Energetic Materials. Propellants Explos. Pyrotech. **40**, 366–373. (10.1002/prep.201400294)

[31] Popova EA, Trifonov RE, Ostrovskii VA. 2011 Advances in synthesis of tetrazoles coordinated to metal ions. Arkivoc **2012**, 45–65. (10.3998/ark.5550190.0013.102)

[32] Tamura Y *et al*. 1998 Highly Selective and Orally Active Inhibitors of Type IV Collagenase (MMP-9 and MMP-2): N -Sulfonylamino Acid Derivatives. J. Med. Chem. **41**, 640–649. (10.1021/jm9707582) 9484512

[33] Wood E *et al*. 2001 A prodrug approach to the design of cRaf1 kinase inhibitors with improved cellular activity. Anticancer Drug Des. **16**, 1–6. 11762640

[34] Meanwell NA. 2011 Synopsis of Some Recent Tactical Application of Bioisosteres in Drug Design. J. Med. Chem. **54**, 2529–2591. (10.1021/jm1013693) 21413808

[35] Malik MA, Wani MY, Al-Thabaiti SA, Shiekh RA. 2014 Tetrazoles as carboxylic acid isosteres: chemistry and biology. J. Incl. Phenom. Macrocycl. Chem. **78**, 15–37. (10.1007/s10847-013-0334-x)

[36] Mittal R, Awasthi SK. 2019 Recent Advances in the Synthesis of 5-Substituted 1H-Tetrazoles: A Complete Survey (2013–2018). Synthesis **51**, 3765–3783. (10.1055/s-0037-1611863)

[37] Baskaya G, Esirden İ, Erken E, Sen F, Kaya M. 2017 Synthesis of 5-Substituted-1H-Tetrazole Derivatives Using Monodisperse Carbon Black Decorated Pt Nanoparticles as Heterogeneous Nanocatalysts. J. Nanosci. Nanotechnol. **17**, 1992–1999. (10.1166/jnn.2017.12867)

[38] Wang H, Wang Y, Han Y, Zhao W, Wang X. 2020 Humic acid as an efficient and reusable catalyst for one pot three-component green synthesis of 5-substituted 1 H -tetrazoles in water. RSC Adv. **10**, 784–789. (10.1039/c9ra08523h) 35494449 PMC9047532

[39] Amantini D, Beleggia R, Fringuelli F, Pizzo F, Vaccaro L. 2004 TBAF-Catalyzed Synthesis of 5-Substituted 1H -Tetrazoles under Solventless Conditions. J. Org. Chem. **69**, 2896–2898. (10.1021/jo0499468) 15074950

[40] Matsugi M, Ishihara K, Kawashima M, Shioiri T. 2016 Synthesis of 5-Substituted 1H-Tetrazoles from Aldoximes Using Diphenyl Phosphorazidate. Synlett **27**, 2225–2228. (10.1055/s-0035-1561668)

[41] Carpentier F, Felpin FX, Zammattio F, Le Grognec E. 2020 Synthesis of 5-Substituted 1H -Tetrazoles from Nitriles by Continuous Flow: Application to the Synthesis of Valsartan. Org. Process Res. Dev. **24**, 752–761. (10.1021/acs.oprd.9b00526)

[42] Rama V, Kanagaraj K, Pitchumani K. 2011 Syntheses of 5-Substituted 1 H -Tetrazoles Catalyzed by Reusable CoY Zeolite. J. Org. Chem. **76**, 9090–9095. (10.1021/jo201261w) 21910486

[43] Mitra B, Mukherjee S, Pariyar GC, Ghosh P. 2018 One pot three-component synthesis of 5-substituted 1 H -tetrazole from aldehyde. Tetrahedron Lett. **59**, 1385–1389. (10.1016/j.tetlet.2018.02.067)

[44] Alotaibi MR, Monier M, Elsayed NH. 2020 Fabrication and investigation of gold (III) ion‐imprinted functionalized silica particles. J. Mol. Recognit. **33**, e2813. (10.1002/jmr.2813) 31814208

[45] Tarade K, Shinde S, Rode C. 2020 Magnetically separable catalyst for condensation of renewable aldehydes and 2-methylfuran to saturated cyclic oxygenates. Fuel Process. Technol. **197**, 106191. (10.1016/j.fuproc.2019.106191)

[46] Ocello R, De Nisi A, Jia M, Yang Q, Monari M, Giacinto P, Bottoni A, Miscione GP, Bandini M. 2015 Gold(I)‐Catalyzed Dearomative [2+2]‐Cycloaddition of Indoles with Activated Allenes: A Combined Experimental–Computational Study. Chem. Eur. J. **21**, 18445–18453. (10.1002/chem.201503598) 26517191

[47] Sithole RK, Machogo LFE, Airo MA, Gqoba SS, Moloto MJ, Shumbula P, Van Wyk J, Moloto N. 2018 Synthesis and characterization of Cu3N nanoparticles using pyrrole-2-carbaldpropyliminato Cu(II) complex and Cu(NO3)2. New J. Chem. **42**, 3042–3049. (10.1039/c7nj05181f)

[48] Pirani F, Eshghi H, Rounaghi SA. 2023 Immobilized Cu(0) nanoparticles on montmorillonite-modified with benzalkonium chloride (MMT-BAC@Cu(0)): as an eco-friendly and proficient heterogeneous nano-catalyst for green synthesis of 5-substituted 1 H -tetrazoles. RSC Adv. **13**, 6160–6170. (10.1039/d2ra08208j) 36814874 PMC9940308

[49] Esmaeilpour M, Sardarian AR, Firouzabadi H. 2018 Dendrimer‐encapsulated Cu(Π) nanoparticles immobilized on superparamagnetic Fe_3_O_4_@SiO_2_ nanoparticles as a novel recyclable catalyst for N-arylation of nitrogen heterocycles and green synthesis of 5-substituted 1H-tetrazoles. Appl. Organomet. Chem. **32**, 4300. (10.1002/aoc.4300)

[50] Gavrilov M, Zerk TJ, Bernhardt PV, Percec V, Monteiro MJ. 2016 SET-LRP of NIPAM in water via in situ reduction of Cu(ii) to Cu(0) with NaBH4. Polym. Chem. **7**, 933–939. (10.1039/c5py01855b)

[51] Sharma C, Kaur M, Choudhary A, Sharma S, Paul S. 2020 Nitrogen Doped Carbon–Silica Based Cu(0) Nanometal Catalyst Enriched with Well-Defined N-moieties: Synthesis and Application in One-Pot Synthesis of 1,4-Disubstituted-1,2,3-triazoles. Catal. Lett. **150**, 82–94. (10.1007/s10562-019-02936-y)

[52] Aromaa J, Kekkonen M, Mousapour M, Jokilaakso A, Lundström M. 2021 The Oxidation of Copper in Air at Temperatures up to 100 °C. CMD **2**, 625–640. (10.3390/cmd2040033)

[53] Asfaram A, Ghaedi M, Agarwal S, Tyagi I, Kumar Gupta V. 2015 Removal of basic dye Auramine-O by ZnS:Cu nanoparticles loaded on activated carbon: optimization of parameters using response surface methodology with central composite design. RSC Adv. **5**, 18438–18450. (10.1039/c4ra15637d)

[54] Rostovtsev VV, Green LG, Fokin VV, Sharpless KB. 2002 A Stepwise Huisgen Cycloaddition Process: Copper(I)-Catalyzed Regioselective ‘Ligation’ of Azides and Terminal Alkynes. Angew. Chem. Int. Ed. **41**, 2708–2711. (10.1002/1521-3773(20020715)41:143.0.co;2-4) 12203546

[55] Himo F, Lovell T, Hilgraf R, Rostovtsev VV, Noodleman L, Sharpless KB, Fokin VV. 2005 Copper(I)-Catalyzed Synthesis of Azoles. DFT Study Predicts Unprecedented Reactivity and Intermediates. J. Am. Chem. Soc. **127**, 210–216. (10.1021/ja0471525) 15631470

[56] Arefi E, Khojastehnezhad A, Shiri A. 2021 A magnetic copper organic framework material as an efficient and recyclable catalyst for the synthesis of 1,2,3-triazole derivatives. Sci. Rep. **11**, 20514. (10.1038/s41598-021-00012-3) 34654831 PMC8519936

[57] Kumar A, Verma S, Pathak DD. 2021 Synthesis and characterization of a recyclable graphene oxide-surface- engineered copper(II) Schiff base complex: Catalytic application in synthesis of 1,2,3-triazoles and 2H-indazoles. J. Environ. Chem. Eng. **9**, 105791. (10.1016/j.jece.2021.105791)

[58] Kazemnejadi M, Sardarian AR. 2016 Ecofriendly synthesis of a heterogeneous polyvinyl alcohol immobilized copper(ii) Schiff base complex as an efficient, reusable catalyst for the one-pot three-component green preparation of 5-substituted 1H-tetrazoles under mild conditions. RSC Adv. **6**, 91999–92006. (10.1039/c6ra19631d)

[59] Ahmadi A, Sedaghat T, Motamedi H, Azadi R. 2020 Anchoring of Cu (II)‐Schiff base complex on magnetic mesoporous silica nanoparticles: catalytic efficacy in one‐pot synthesis of 5‐substituted‐1H‐tetrazoles, antibacterial activity evaluation and immobilization of α‐amylase. Appl. Organomet. Chem. **34**, 5572. (10.1002/aoc.5572)

[60] Layek S, Agrahari B, Dey S, Ganguly R, Pathak DD. 2019 Copper(II)-faciliated synthesis of substituted thioethers and 5-substituted 1H-tetrazoles: Experimental and theoretical studies. J. Organomet. Chem. **896**, 194–206. (10.1016/j.jorganchem.2019.06.008)

[61] Amiri M, Bagherzadeh N, Sardarian A. 2025 Supplementary material from: Novel, Nanomagnetic, and Recoverable Copper(0)Catalyst in One-Pot Access to 1,4-Disubstituted 1,2,3-Triazoles and 5-Substituted 1H-Tetrazoles in Water. FigShare (10.6084/m9.figshare.c.7618820) PMC11814627 39944294

